# Integrative lipidomic and genomic analysis identifies key lipids for wooden breast in chickens

**DOI:** 10.1186/s40104-026-01517-5

**Published:** 2026-11-03

**Authors:** Yanru Chen, Lu Bai, Xiaochun Ma, Mengjie Wang, Yaoyao Tang, Zhiqing Li, Yingying Pei, Haohan Zhang, Guiping Zhao, Jie Wen, Ranran Liu

**Affiliations:** 1 State Key Laboratory of Animal Biotech Breeding, Institute of Animal Science, Chinese Academy of Agricultural Sciences, The Broiler Research Center of the Chinese Academy of Agricultural Sciences, Beijing, 100193 China; 2 College of Animal Science, South China Agricultural University, Guangzhou, 510642 China; 3 College of Animal Science and Technology, Jilin Agricultural University, Jilin, 130118 China

**Keywords:** Lipidomics, Mediation analysis, Mendelian randomization, mGWAS, Wooden breast

## Abstract

**Background:**

Wooden breast (WB) is a prevalent myopathy in broilers that substantially reduces meat quality and causes considerable economic losses. However, the genetic architecture and lipidomic alterations associated with WB remain poorly understood. This study aimed to integrate untargeted lipidomics and genomic analyses to characterize lipid alterations and their associated genetic factors in WB.

**Results:**

To characterize WB-related phenotypes, pectoralis major muscles from 312 broilers were evaluated for WB severity, compression force (CF), endomysium thickness, and total lipid abundance. By integrating differential abundance analysis, trend analysis, and weighted gene co-expression network analysis (WGCNA), we identified a core set of 25 lipid species. Further refinement using Mendelian randomization (MR) and mediation analyses prioritized nine lipids as candidate molecular traits associated with genetic variation and WB, seven of which showed consistent associations in an independent cohort (*n* = 119). Metabolome genome‐wide association study (mGWAS) of these lipids identified a consensus genetic locus on chromosome 6 (19.55–20.15 Mb) shared by three candidate lipids. Integration of transcriptomic profiles from genes within this locus prioritized five candidate genes, with *MAPK8* and *NPY4R* showing consistent expression patterns in the independent cohort. Based on its potential biological relevance, *MAPK8* was selected for functional characterization in chicken primary myoblasts. *MAPK8* overexpression and knockdown produced opposite effects on lipid accumulation, oxidative stress, and fibrosis-related gene expression, suggesting that *MAPK8* may participate in cellular processes involved in lipid accumulation, oxidative stress, and fibrotic remodeling associated with WB.

**Conclusions:**

This study provides an integrated overview of lipid metabolic alterations and their genetic architecture in WB-affected chickens. Collectively, these findings identify candidate lipid biomarkers and genetic loci and implicate *MAPK8* as a candidate gene associated with lipid deposition and fibrotic remodeling in WB.

**Supplementary Information:**

The online version contains supplementary material available at https://doi.org/10.1186/s40104-026-01517-5.

## Background

As a primary source of animal protein, chicken meat production has increased substantially due to intensive genetic selection for high breast yield. However, this has coincided with the emergence of muscle quality defects, most notably wooden breast (WB) [1]. WB is characterized by hardened pectoralis major muscles and histopathological alterations, including myofiber degeneration, fibrosis, and lipid accumulation, and has been reported in up to 40% of birds within affected flocks [2–4]. These quality defects, marked by poor functional properties and reduced palatability, result in significant consumer rejection and substantial economic losses [5–7].

The etiology of WB is multifactorial, involving an imbalance between accelerated muscle hypertrophy and metabolic demand [8, 9]. Insufficient vascularization may induce localized hypoxia and oxidative stress, triggering metabolic reprogramming and tissue remodeling [10, 11]. These metabolic constraints are proposed to contribute to muscle fiber degeneration, immune cell infiltration, and remodeling processes involving collagen accumulation and lipid deposition [12–14]. While WB is recognized as a moderately heritable trait, the complexity of its genetic architecture has hindered the identification of genetic variants associated with WB [15, 16]. Current nutritional interventions have mitigated severity but failed to eliminate WB, necessitating more integrated approaches to elucidate its underlying mechanisms [17, 18].

Omics technologies have recently revealed extensive metabolic dysregulation in WB [19]. Among these, lipidomics is particularly informative because of lipid infiltration observed in WB and the essential roles of lipid metabolism and signaling in muscle physiology [20]. Previous studies have shown that lipidomic alterations are most pronounced in the breast muscle, characterized by shifts in neutral lipid proportions [21]. Consequently, detailed profiling of the breast muscle lipidome is essential for identifying metabolic pathways perturbed in WB [22–24]. Integrating these data with genomic analyses facilitates the identification of genetic loci associated with lipid traits and improves understanding of the genetic basis of metabolic variation in complex phenotypes [25]. Such multi-omics strategies have proven effective in identifying livestock meat quality biomarkers and unraveling the genetic regulation of complex metabolic processes [26].

In this study, we integrated untargeted lipidomics with genomic analyses to characterize lipid remodeling in WB-affected broilers and identify key lipid species and candidate genes. This work provides insights into the molecular and genetic factors associated with WB.

## Methods

### Animals and sampling

A total of 312 male Guangming No. 2 broilers from two pure broiler lines (159 from line 1 and 153 from line 2) were reared at Xinguang Agriculture and Animal Husbandry Co., Ltd. (Mile, Yunnan, China) under standardized conditions. The two broiler lines share the same background but represent distinct breeding populations. Birds were slaughtered at 42 d of age, with an average body weight of 3.2 ± 0.4 kg, following a 12-h feed withdrawal. Stunning was performed using low-voltage electrical stunning (50 V, 400 Hz, 15 s), followed by exsanguination via neck transection.

Blood was collected from each chicken into EDTA-coated tubes one day before slaughter and refrigerated at 4 °C. Genomic DNA was extracted from whole blood within 24 h using a magnetic-bead method. Within 15 min postmortem, approximately 50 mg of muscle tissue was excised from the mid-upper region of the right pectoralis major of each chicken. Samples were immediately snap-frozen in liquid nitrogen and stored at –80 °C. Whole-genome sequencing was performed at an average depth of 20 × per individual.

### WB scoring and compression force (CF) testing

WB was evaluated in the left pectoralis major of each chicken. Two trained personnel independently performed subjective WB scoring 2 h postmortem, following the criteria of Liu et al. [21]. The scoring scale was 0–3: WB0 (normal breast, soft throughout), WB1 (mild WB, firm only at the anterior end of the muscle), WB2 (moderate WB, firm from the anterior through the mid region, soft posteriorly), and WB3 (severe WB, overall firm texture, often with hemorrhages or exuded fluid).

Mechanical texture was quantified by measuring CF in the pectoralis major muscle two days postmortem. Each muscle was placed flat on the stage of a TA.XT Plus Texture Analyzer (Stable Micro Systems, UK). A 6-mm-diameter flat-ended cylindrical probe (P/6) was used to compress the muscle to 20% of its original height. The instrument was set to a trigger force of 5 g, pre- and post-test speeds of 10 mm/s, and a test speed of 5 mm/s. A single compression was applied at the highest point of the breast, and the maximum force recorded was defined as the CF value for each sample.

### Histological staining and image analysis

After removal of the overlying skin from the upper pectoral muscle, tissue strips measuring approximately 1 cm × 1 cm × 0.5 cm were dissected from the same region of each breast sample. The tissues were fixed in 4% paraformaldehyde solution for 48 h, dehydrated, and embedded in paraffin. Serial cross-sections (5 μm thick) were prepared using a rotary microtome perpendicular to the orientation of the muscle fibers.

Picrosirius Red staining was performed to visualize collagen fiber morphology following Rittié [27] with minor modifications. Stained slides were imaged under bright-field illumination using an Olympus BX-series microscope. Images were acquired using OlyVIA software (v4.1.27564, Olympus, Japan). Hematoxylin–eosin (HE) staining was conducted on parallel sections. Images were acquired using an Olympus BX51 microscope, and six non-overlapping fields per sample were analyzed. Endomysium thickness, defined as the distance between adjacent myofibers within a bundle, was measured 100 times per section and averaged using Image-Pro Plus (v6.0).

### Population structure analysis

Principal component analysis (PCA) was performed using genome-wide single nucleotide polymorphism (SNP) markers. Before PCA, SNP markers were subjected to linkage disequilibrium (LD) pruning using PLINK v1.9 with the parameters --indep-pairwise 50 5 0.2. PCA was then conducted using the LD-pruned SNP set (--pca) in PLINK. The first two principal components were plotted by breeding line to assess population structure.

### Lipid extraction and HPLC–MS analysis

Lipids were extracted using a modified organic solvent extraction protocol [28]. Briefly, approximately 50 mg frozen tissue was homogenized in 300 µL cold methanol containing UltimateSPLASH™ ONE internal standards (Sigma-Aldrich) using bead-beating (60 s). Subsequently, 600 µL of dichloromethane was added, and the mixture was vortexed for 20 s. Phase separation was induced by adding 270 µL of ultrapure water twice, followed by centrifugation at 5,000 r/min for 10 min. The lower organic phase was collected, dried under a nitrogen stream, and stored at −80 °C. Extracts were reconstituted in dichloromethane:methanol (1:1, v/v) prior to analysis.

Quality control procedures included blank extractions and pooled QC samples to monitor analytical stability, while stearic acid (C18:0) and eicosapentaenoic acid (EPA) were used as recovery standards. Lipid recovery was greater than 85% [29].

Chromatographic separation was performed on an ExionLC™ AD system (AB Sciex) using an ACQUITY UPLC HSS T3 column (2.1 mm × 100 mm, 1.8 µm; Waters) at 40 °C. Mobile phase A consisted of acetonitrile:water (60:40, v/v) with 10 mmol/L ammonium formate, and mobile phase B consisted of acetonitrile:isopropanol (10:90, v/v) with 10 mmol/L ammonium formate. The gradient elution was 100% A (0–1 min), 50% A (1–6 min), 0% A (6–30 min), held at 0% A (30–32 min), and re-equilibrated to 100% A (32.1–35 min) at 0.3 mL/min.

Mass spectrometry was conducted on a TripleTOF^®^ 6600 system (AB Sciex, Framingham, MA, USA) equipped with an electrospray ionization (ESI) source. The source parameters were optimized as follows: Ion Source Gas 1 (GS1): 50 psi; Ion Source Gas 2 (GS2): 50 psi; Curtain Gas (CUR): 35 psi; Source Temperature (TEM): 500 °C. The ion spray voltage floating (ISVF) was set at 5500 V for positive mode and –4500 V for negative mode. The declustering potential (DP) was ±100 V. Data were acquired using data-dependent acquisition (DDA) mode. In positive ion mode, the scan ranges were *m/z* 300–1,200 (MS1) and *m/z* 50–1,200 (MS2). For the negative mode, the scan ranges were *m/z* 100–1,200 (MS1) and *m/z* 50–1,200 (MS2). The collision energy (CE) was set to 35 ± 15 eV.

Raw data were processed using MS-DIAL and annotated against LipidBlast, HMDB, GNPS, METLIN, MoNA, and an in-house library. Lipids were identified based on retention time (± 0.2 min) and MS/MS similarity score > 80%.

Relative lipid abundances were obtained from normalized peak areas and used for all downstream analyses, including differential abundance analysis, weighted gene co-expression network analysis (WGCNA), metabolome genome‐wide association study (mGWAS), Mendelian randomization (MR), and mediation analyses. Total lipid abundance was calculated as the sum of the normalized peak areas of all annotated lipid species in each sample and expressed in arbitrary units (a.u.) as a relative measure of overall lipid abundance. Features with a relative standard deviation (RSD) > 30% among pooled QC samples were excluded from downstream analyses.

### Heritability estimation of lipid traits

Genomic heritability (*h*^2^) of lipid abundance was estimated using the LDAK-thin model implemented in LDAK v6.1 [30]. Single-component mixed models were fitted separately for SNPs, insertions/deletions (InDels), and structural variants (SVs). For each variant class, variants were LD-pruned using the LDAK-thin procedure (--window-prune 0.98 --window-kb 100), and genetic relationship matrices were constructed using the LDAK weighting scheme with the recommended scaling parameter (α = −0.25). Population structure was controlled for by including the first three principal components calculated with PLINK, together with broiler line, as fixed-effect covariates. *h*^2^ estimates were constrained to the interval [0,1]. Only converged models were retained for downstream analyses. Mean genomic *h*^2^ and the corresponding mean model-estimated standard error (SE) were summarized across the 1,003 lipid species for each variant class.

### Fatty acid composition analysis

Fatty acid composition was inferred from annotated lipid molecular species based on their acyl chain information. Structural lipids containing identifiable fatty acid chains were classified according to saturation status (SFA, MUFA, and PUFA) and chain length (SCFA/MCFA, LCFA, and VLCFA). For each lipid species, chain abundance was weighted by its measured relative abundance and the number of corresponding fatty acid chains. Sample-level fatty acid composition was calculated by summing weighted abundance of individual lipid species within each category. Differences in PUFA/SFA ratios between groups were evaluated using the Wilcoxon rank-sum test with multiple testing correction.

### Differential abundance, dynamic trend, and co-expression network analysis

Differential lipid abundance analysis was performed using the limma R package. Lipid abundances were log₂-transformed and fitted to a linear model using a design matrix representing four WB scores. Empirical Bayes moderation was applied, and pairwise contrasts were constructed between the WB0 group and the WB1 + 2 + 3 group, in which WB1, WB2, and WB3 samples were pooled into a single WB-affected group. To control the false positive rate, *P* values were adjusted using the Benjamini–Hochberg False Discovery Rate (FDR) method applied across all 1,003 detected lipids within each pairwise contrast. Differentially abundant lipids were defined as those with FDR < 0.05 and |log₂FC| ≥ 1.

Lipid dynamics across WB scores were evaluated using linear regression with WB score treated as a continuous variable. *P* values were adjusted using the Benjamini–Hochberg method. Lipids with significant trends (FDR < 0.05) were Z-score normalized and clustered using K-means (k = 4).

Weighted co-expression network analysis was performed using the WGCNA R package [31]. Lipid abundances were log-transformed and standardized, and outlier samples were checked via hierarchical clustering. A weighted adjacency matrix was constructed based on Pearson correlations and transformed using a soft-thresholding power *β* to achieve scale-free topology (*R*^2^ > 0.8) [32–34]. The resulting adjacency matrix was converted into a topological overlap matrix (TOM), and lipids were clustered into modules using a dynamic tree-cut algorithm. Highly correlated modules were merged based on eigengene correlations. Module eigengenes were correlated with phenotypic traits using Pearson correlation analysis (*P* < 0.05).

### MR and mediation analysis

MR was performed using genetic variants as instrumental variables (IVs). For each lipid, variants associated at *P* < 1 × 10⁻^5^ in the mGWAS were selected. LD clumping was conducted using PLINK with *r*^2^ < 0.1 within a 500-kb window based on the study population genotype. Instrument strength was evaluated using the *F*-statistic (*F* = *β*^2^/SE^2^), and only lipids with mean *F* > 10 were retained.

Exposure and outcome datasets were harmonized to align effect alleles, and palindromic variants with intermediate allele frequencies were removed. For lipid traits instrumented by a single variant, the Wald ratio method was applied; for two variants, the inverse variance weighted (IVW) method was used; and for three or more variants, both IVW and MR-Egger regression were performed. IVW estimates were considered the primary MR estimates. Horizontal pleiotropy was evaluated using the MR-Egger intercept test, whereas heterogeneity among instrumental variables was assessed using Cochran's Q statistic. For lipid traits instrumented by three or more variants, leave-one-out analysis was additionally performed. Sensitivity analyses were further conducted using more stringent IV selection thresholds (*P* < 1.1 × 10⁻⁶ and *P* < 5.48 × 10⁻⁸).

Lipid traits showing evidence of association in the initial MR analysis (IVW *P* < 0.05) were subsequently evaluated using a two-step MR mediation framework [35]. In step one, the genetic effect on lipid exposure (*β*_XM) was estimated. In step two, a second univariable MR analysis was performed to estimate the association between lipid traits and WB outcome (*β*_MY). The indirect effect was calculated as *β*_XM × *β*_MY, with its standard error estimated using the Delta method. Significance was assessed using a Z-test, and the mediated proportion was calculated as (indirect effect/*β*_XY). Lipids showing significant indirect effects (*P* < 0.05) were prioritized as candidate genetically supported intermediate traits. For validation, genotypes of significant IVs were extracted using PLINK (--recode A) and integrated with phenotypic and lipid abundance data.

### mGWAS and identification of candidate genetic regions

A total of 12,869,343 genomic variants, including SNPs, InDels, and SVs, were used for association analysis. Single-trait genome-wide association tests were performed using the mixed linear model (MLM) implemented in GCTA (v1.94), specified as *y* = *Wα* + *xβ* + *u* + *ε*, where *y* is the vector of log-transformed lipid abundances, *W* represents fixed effects, including the intercept and line (two broiler lines, fitted as a categorical covariate), *x* is the genotype vector, *β* is the variant effect, *u* ~ *MVN(0, λτ*^–1^*K)* denotes random polygenic effects based on the genomic relationship matrix (*K*), and *ε* ~ *MVN(0, τ*^–1^*I)* is the residual error. The genomic relationship matrix (*K*) was constructed from all quality-controlled autosomal variants using the --make-grm --autosome function in GCTA, without LD pruning.

To account for LD, the effective number of independent tests was estimated using PLINK with LD pruning (--indep-pairwise 25 5 0.2) applied to the SNP dataset [36]. SNPs were used because they constitute the predominant class of genomic variants and provide a well-established basis for estimating the number of effectively independent tests. This yielded 913,081 independent tests (*M*ₑff). Accordingly, genome-wide significance threshold was set at *P* < 5.48 × 10^–8^ (0.05/*M*ₑff; − log₁₀*P* = 7.26), and the suggestive threshold at *P* < 1.10 × 10^–6^ (1/*M*ₑff; − log₁₀*P* = 5.96).

For prioritized lipids, genome-wide significant variants were extracted and assigned to their corresponding traits. Lead variants were identified separately for each variant class using PLINK clumping analysis with an LD threshold of *r*^*2*^ < 0.6 within a 500-kb window. LD-associated variants were subsequently identified using PLINK with *r*^2^ ≥ 0.6 within a 1-Mb window, and the genomic coordinates of these variants were used to define lipid-specific candidate intervals. Overlapping candidate intervals across lipid traits and variant classes were defined as consensus genetic hotspots.

### Receiver operating characteristic (ROC) analysis

To evaluate the classification performance of candidate lipids for distinguishing WB from normal muscle, ROC analysis was performed using the pROC package in R. Multivariate classification models were constructed using generalized linear models with a binomial logit link function. In the discovery cohort (*n* = 312), 10-fold cross-validation was applied to estimate internal model performance and reduce overfitting. Cross-validated predicted probabilities were used to generate ROC curves and calculate AUC values with 95% confidence intervals. DeLong’s test was used to compare AUCs between ROC models.

To assess model generalizability, an independent validation cohort (*n* = 119) was used for external validation. The final 7-lipid panel was trained in the discovery cohort, and the optimal classification threshold was determined only in the discovery cohort using the Youden index. This threshold was then fixed and applied directly to the validation cohort without re-optimization. Diagnostic performance in the validation cohort was evaluated by AUC, accuracy, sensitivity, specificity, positive predictive value (PPV), negative predictive value (NPV), and F1 score, all derived from the confusion matrix. All statistical analyses were performed in R (v4.5.1) using the pROC and caret packages.

### RNA extraction and sequencing

Total RNA was extracted from pectoralis major muscle samples using TRIzol reagent (Invitrogen). RNA quality was assessed using a NanoDrop 2000 spectrophotometer (Thermo Scientific, USA) and an Agilent 2100 Bioanalyzer (Agilent Technologies, USA). Only samples with RNA integrity number > 7.0 were used for library construction. Sequencing libraries were prepared using the NEBNext Ultra RNA Library Prep Kit for Illumina and sequenced on the Illumina NovaSeq 6000 platform with 150-bp paired-end reads.

Raw reads were filtered to remove adapters and low-quality sequences using fastp (version 0.23.2). The resulting reads were mapped to the chicken reference genome GRCg7b using HISAT2 (version 2.2.1). Gene-level read counts were generated using featureCounts and normalized to transcripts per million (TPM).

### Independent cohort validation of transcriptome and lipidome

To validate the multi-omics findings, an independent validation cohort consisting of 119 broilers was used, including 81 WB0, 26 WB1, 7 WB2, and 5 WB3 individuals. Muscle tissues were collected using the same standardized procedures as the discovery cohort. Total RNA extraction, RNA sequencing, and untargeted lipidomic profiling were performed using the same protocols, and the resulting data were processed using the same bioinformatics pipelines as described above. Owing to the limited number of WB2 and WB3 cases, most validation analyses were performed by comparing WB0 and WB1 + 2 + 3 birds. Endomysium thickness data were available for 27 birds (12 WB0 and 15 WB1 + 2 + 3) in the validation cohort.

### Isolation and culture of chicken primary myoblasts

According to previously published methods with minor modifications, chicken primary myoblasts were isolated from breast muscle tissues of 14-day-old chicken embryos [37, 38]. Briefly, the tissues were minced and digested with collagenase and trypsin. The isolated cells were filtered, centrifuged, and seeded into culture dishes. Cells were maintained in a growth medium (DMEM/F12) supplemented with 20% fetal bovine serum (FBS) and 1% penicillin–streptomycin in a humidified incubator at 37 °C with 5% CO₂. Myoblasts were purified using the differential adhesion method [38].

### Plasmid and siRNA transfection

To investigate the biological function of *MAPK8* in chicken primary myoblasts, a *MAPK8* overexpression plasmid (OE-*MAPK8*, GenePharma, Shanghai, China) and a specific small interfering RNA (si-*MAPK8*, GenePharma, Shanghai, China) were used. The *MAPK8* coding sequence was cloned into the pcDNA3.1(+)−3 × FLAG expression vector. The sequence of the *MAPK8* siRNA was 5′-AUCCAUCUUCGUCGUCAUCTT-3′. When myoblasts reached approximately 70%–80% confluence, cells were transfected according to the manufacturers' instructions. The *MAPK8* overexpression plasmid and corresponding empty vector were transfected using Lipofectamine™ 3000 (Invitrogen, Carlsbad, CA, USA), whereas the *MAPK8* siRNA and negative control siRNA (siNC) were transfected using siRNA-Mate SUS Transfection Reagent (GenePharma, Shanghai, China). At 24 h after transfection, the growth medium was replaced with myogenic differentiation medium consisting of high-glucose DMEM supplemented with 2% horse serum. Cells were differentiated for an additional 24 h before RNA extraction or 48 h before protein extraction and fluorescence staining.

### RNA extraction and real-time quantitative PCR (qPCR)

Total RNA was extracted from transfected chicken primary myoblasts using TRIzol reagent (Invitrogen, Carlsbad, CA, USA) according to the manufacturer's instructions. RNA was reverse-transcribed into complementary DNA (cDNA) using a reverse transcription kit. qPCR was performed using PowerUp™ SYBR™ Green Master Mix (Applied Biosystems, Foster City, CA, USA) on a QuantStudio™ real-time PCR system (Applied Biosystems). Primer sequences were designed based on the *Gallus gallus* reference sequences deposited in the NCBI GenBank database. The primer sequences, corresponding GenBank accession numbers, and amplicon sizes are provided in Table 1. Relative mRNA expression levels were calculated using the 2^−ΔΔCt^ method, with *GAPDH* as the endogenous reference gene. Each sample was analyzed in three technical replicates.

**Table 1:** Primer sequences used for quantitative PCR

Gene	GenBank accession number^a^	Primer sequence (5′→3′)	Orientation	Amplicon size, bp
*MAPK8*	XM_040703166.1	TCCATCAGAAGCCGAAGCTC	Forward	177
		CATTGCTGCACCTGTGCTAA	Reverse	
*PPARγ*	NM_001001460.2	TGACAGCGCCAGAGATTACA	Forward	93
		CATCCATCGCAGACAGATCCA	Reverse	
*COL1A1*	NM_001396622.1	ATCAGGGCCGAAGGAAACAG	Forward	72
		TGCTCCAGTGTGACTCGTG	Reverse	
*COL1A2*	NM_001079714.2	TGCGTCCTGCAGGTAATAGC	Forward	158
		GCCTCACTCACATGTTGGCT	Reverse	
*TGFB1*	NM_001318456.1	CAGCAGCCGGGGGAATC	Forward	90
		ATAACTCCGATGGGGCACCG	Reverse	
*GAPDH*	NM_204305.2	TCGGAGTCAACGGATTTGGC	Forward	181
		TTCCCGTTCTCAGCCTTGAC	Reverse	

*MAPK8* Mitogen-activated protein kinase 8, *PPARγ* Peroxisome proliferator-activated receptor gamma, *COL1A1* Collagen type I alpha 1 chain, *COL1A2* Collagen type I alpha 2 chain, *TGFB1* Transforming growth factor beta 1, *GAPDH* Glyceraldehyde-3-phosphate dehydrogenase

^a^GenBank accession numbers refer to sequences deposited in the NCBI GenBank database

### Western blot analysis

Total protein was extracted from chicken primary myoblasts using RIPA lysis buffer supplemented with a protease inhibitor cocktail. Protein lysates were mixed with 5 × SDS loading buffer, denatured at 95 °C for 10 min, separated by 10% SDS–PAGE, and transferred onto PVDF membranes. After blocking with 5% non-fat milk in TBST for 1 h at room temperature, membranes were incubated overnight at 4 °C with rabbit anti-MAPK8 primary antibody (Proteintech, Cat. No. 24164-1-AP) and rabbit anti-GAPDH primary antibody (Proteintech, Cat. No. 10494-1-AP). After washing with TBST, membranes were incubated with HRP-conjugated goat anti-rabbit IgG secondary antibody (Proteintech, Cat. No. SA00001-2) for 1 h at room temperature. Protein bands were visualized using an enhanced chemiluminescence (ECL, BIO-RAD, Clarity™ Western ECL Substrate) detection reagent and imaged with a chemiluminescence imaging system. The MAPK8 antibody recognizes both endogenous MAPK8 and the exogenous 3 × FLAG-tagged MAPK8 fusion protein expressed from the overexpression vector.

### Measurement of reactive oxygen species (ROS)

Intracellular ROS levels were determined using the DCFH-DA fluorescent probe (Beyotime Biotechnology, Shanghai, China) [39]. Chicken primary myoblasts were cultured in 20-mm glass-bottom confocal dishes and transfected as described above. Following the designated differentiation period, cells were washed twice with serum-free DMEM and incubated with DCFH-DA working solution (10 μmol/L) at 37 °C for 20 min in the dark. After three washes with serum-free DMEM to remove excess probe, intracellular ROS fluorescence was observed using a fluorescence microscope (excitation wavelength: 488 nm; emission wavelength: 525 nm). Fluorescence intensity was quantified using ImageJ as an indicator of intracellular oxidative stress.

### BODIPY staining and confocal microscopy

To evaluate intracellular lipid accumulation, transfected chicken primary myoblasts cultured in 20-mm glass-bottom confocal dishes were fixed with 4% paraformaldehyde for 15 min at room temperature and washed three times with PBS. Cells were incubated with BODIPY 493/503 (Glpbio, Cat. No. GC42959) in the dark to stain neutral lipid droplets, followed by nuclear counterstaining with DAPI [40]. Fluorescence images were acquired using a Leica TCS SP8 laser scanning confocal microscope (Leica Microsystems, Wetzlar, Germany). Lipid droplet number and area per cell were quantified using ImageJ.

### Statistical analysis

All data were tested for normality using the Shapiro–Wilk test and for homogeneity of variances using Levene's test prior to analysis. Details of statistical analyses, including statistical tests, numbers of replicates, and precision measures, are provided in the corresponding figure legends. To account for differences between the two broiler lines, line was included as a covariate in all differential abundance analyses. The Line × WB interaction was assessed using a two-factor linear model implemented in limma. All analyses were performed in R (v4.5.1).

## Results

### Phenotypic and histological characteristics of WB

Among 312 chickens, 152 were classified as WB0, 75 as WB1, 42 as WB2, and 43 as WB3. Line 1 (*n* = 159) included 115 WB0, 18 WB1, 16 WB2, and 10 WB3 individuals, while Line 2 (*n* = 153) included 37 WB0, 57 WB1, 26 WB2, and 33 WB3 individuals. The distribution of WB severity differed significantly between two broiler lines (χ^2^ = 74.902, *P* < 0.001). Line 1 contained a substantially higher proportion of WB0 samples, whereas Line 2 exhibited a greater proportion of WB2 and WB3 samples (Fig. S1A). Principal component analysis based on genome-wide SNPs revealed clear separation between the two broiler lines, indicating genetic differentiation between populations (Fig. S1B).

**Fig. 1:**
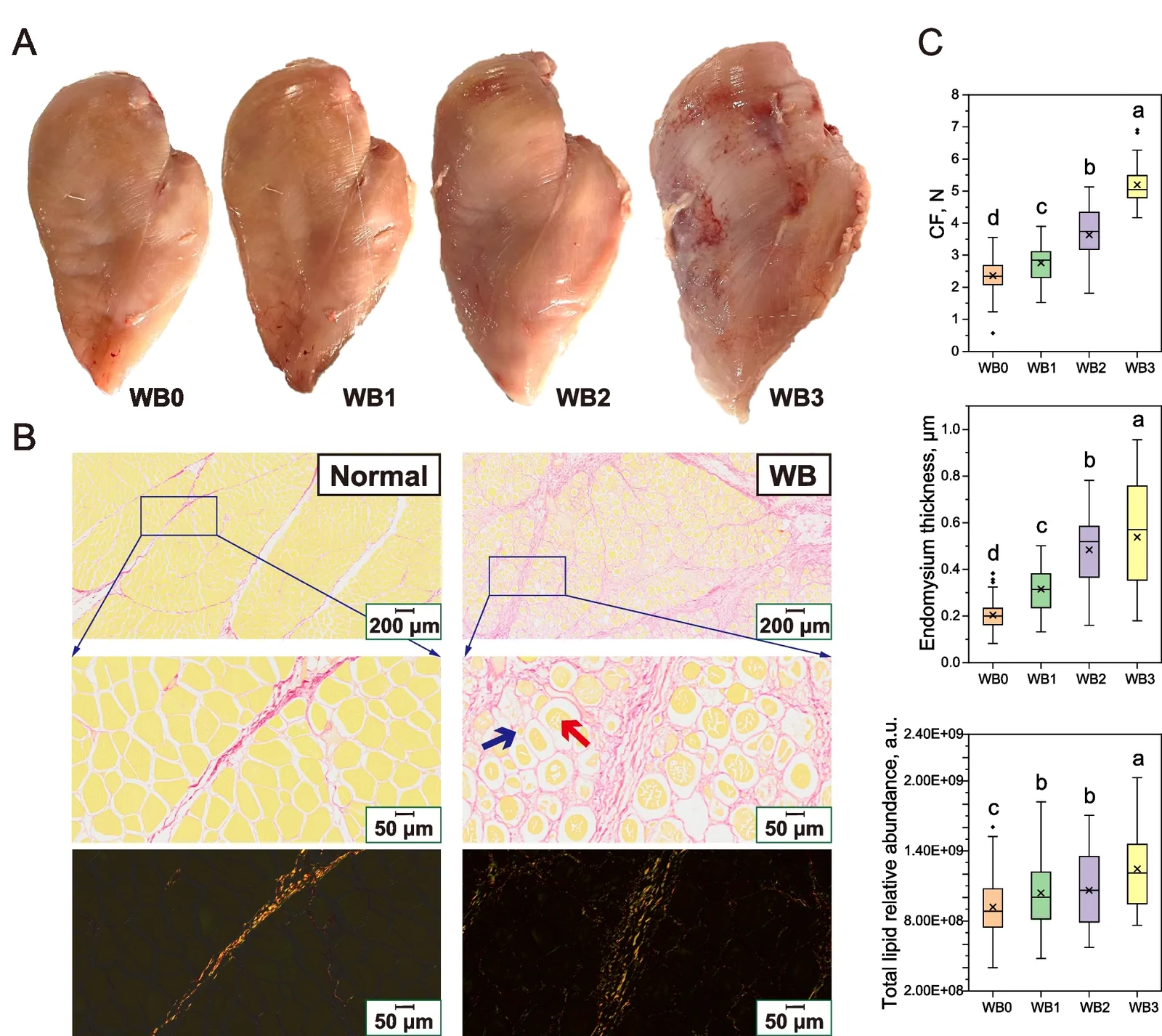
Phenotypic and histological characteristics of WB. **A** Representative macroscopic photographs of breast muscles across four scores.** B** Picrosirius Red staining of normal and WB muscles under bright-field and polarized light. Type I collagen appears yellow/red, whereas type III collagen appears green. Blue arrows indicate regenerated myofibers with small diameters, and red arrows indicate vacuolated myofibers. **C** Phenotypic differences across WB scores. Statistical significance was determined using the Kruskal–Wallis test with Dunn’s post-hoc correction. ^a–d^Bars with different lowercase letters above them indicate significant differences (*P* < 0.05)

As shown in Fig. 1A, WB-affected pectoralis major muscles were markedly harder and less elastic than normal muscles. WB1 was characterized by localized cranial hardening, whereas WB2 and WB3 showed progressively expanded affected regions. WB3 samples often exhibited pale bulging surfaces with petechiae, minor hemorrhage, and exudation of viscous fluids.

Histopathological analysis confirmed these changes. HE staining showed that normal muscle fibers had regular morphology with tight organization and narrow perimysial spaces. In contrast, WB muscles exhibited fiber degeneration and necrosis, increased small regenerating fibers, swollen rounded fibers, and extensive vacuolar changes. Perimysial connective tissue was markedly expanded, with increased endomysium thickness (Fig. S2). Picrosirius Red staining revealed densely organized collagen fibers in normal muscle, whereas WB samples showed disrupted and fragmented collagen architecture with mixed type I and type III collagen fibers (Fig. 1B).

Statistical analysis (Fig. 1C; Table 2) showed that CF, endomysium thickness, and total lipid abundance were significantly higher in WB1–WB3 than in WB0 (*P* < 0.05), with a clear severity-dependent increasing trend. Accordingly, these traits, together with WB scores, were considered key WB-associated phenotypes for downstream analyses.

**Table 2:** Descriptive statistics of phenotypic traits across WB scores

Characteristic	WB0	WB1	WB2	WB3	*P* value^2^
	***n* = 152**^**1**^	***n* = 75**^**1**^	***n* = 42**^**1**^	***n* = 43**^**1**^	
CF, N	2.360 ± 0.440	2.764 ± 0.565	3.635 ± 0.798	5.194 ± 0.606	< 0.001
Endomysium thickness, μm	0.204 ± 0.056	0.315 ± 0.088	0.484 ± 0.147	0.538 ± 0.212	< 0.001
Total lipid relative abundance, a.u.	919,171,695 ± 249,466,027	1,040,566,333 ± 306,694,329	1,062,625,833 ± 311,940,633	1,246,165,442 ± 311,207,139	< 0.001

^*1*^Mean ± SD

^*2*^Kruskal-Wallis rank-sum test

### Lipidomic profiling and genetic architecture of breast muscle

**Fig. 2:**
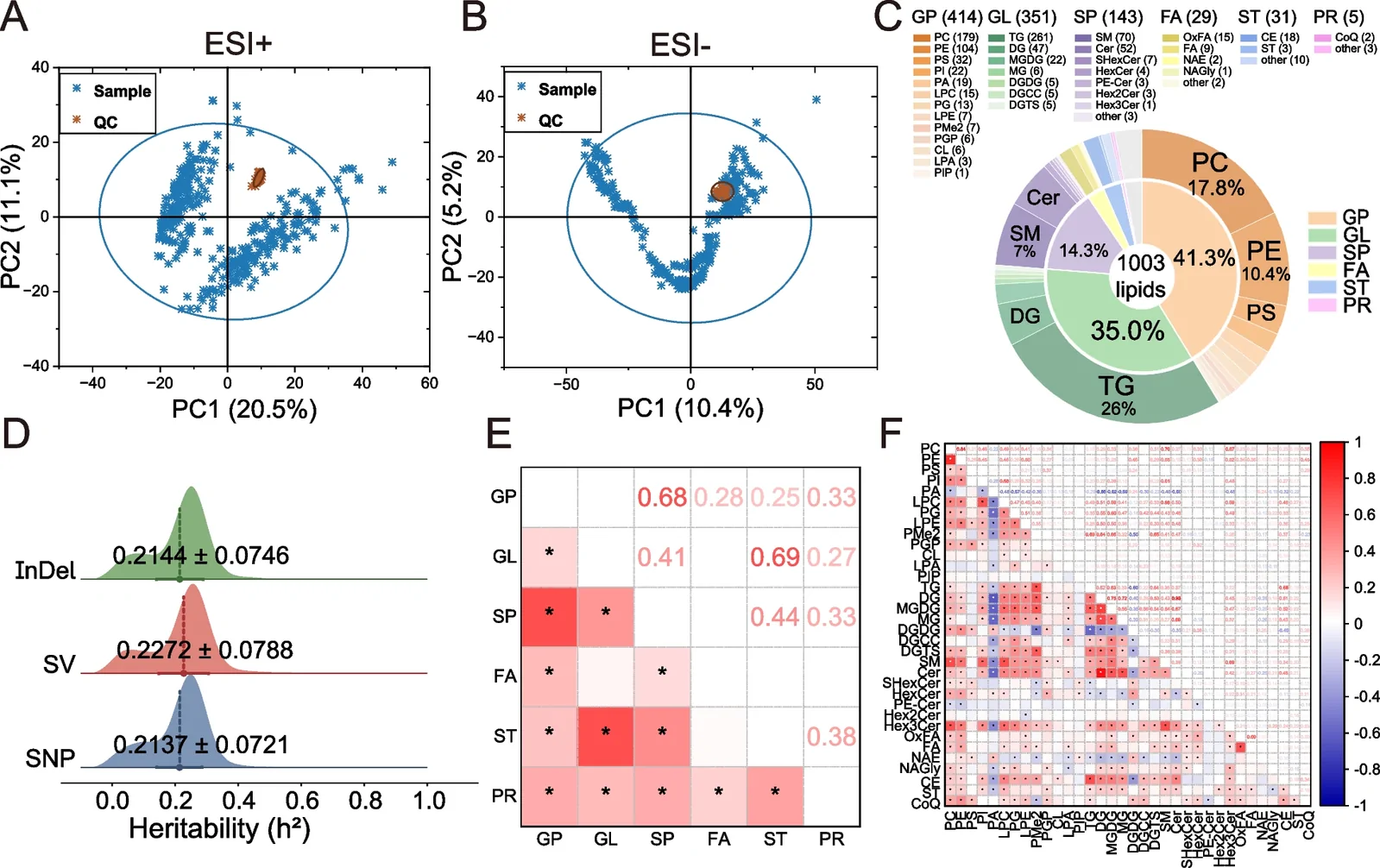
Lipidomic profiling and genetic architecture of breast muscle. **A** and **B** Principal component analysis (PCA) of lipidomic profiles. PCA score plots are shown for positive (ESI +) (**A**) and negative (ESI-) (**B**) electrospray ionization modes. **C** Donut chart illustrates the relative composition of major lipid classes. **D** Ridge plots showing the distribution of genomic heritability (*h*^2^) estimates for 1,003 lipid traits inferred from SNP-, InDel-, and SV-based genomic relationship matrices using the LDAK-thin model. Values displayed on each ridge represent Mean *h*^2^ ± Mean SE across 1,003 lipids. **E** and **F** Pearson correlation analysis among lipid traits. Heatmaps show correlations among major lipid classes (**E**) and lipid subclasses (**F**). Asterisks (*) indicate statistically significant correlations (*P* < 0.05)

1,003 lipid species were identified in breast muscle, including 793 in positive-ion mode and 210 in negative-ion mode. PCA showed tight clustering of pooled quality control samples in both positive electrospray ionization (ESI +) and negative electrospray ionization (ESI −) modes, indicating good analytical stability (Fig. 2A and B). These lipids were annotated into 6 major classes and 34 subclasses, and glycerophospholipids (GPs) were the most abundant class (41.3%), mainly comprising phosphatidylcholines (PCs), phosphatidylethanolamines (PEs), and phosphatidylserines (PSs). Other major subclasses included triacylglycerols (TGs), diacylglycerols (DGs), and sphingomyelins (SMs) (Fig. 2C).

To investigate the genetic architecture of the breast muscle lipidome, genomic *h*^2^ was estimated separately for SNPs, InDels, and SVs. Mean *h*^2^ estimates were 0.2137, 0.2144, and 0.2272, respectively (Fig. 2D). Pairwise Pearson correlations among major lipid classes and subclasses revealed coordinated relationships (Fig. 2E and F). Notably, phospholipid subclasses such as PC, PE, and PS were strongly and positively inter-correlated, reflecting their related biological functions in membrane lipid metabolism.

### Dynamic lipid alterations and their correlations with WB scores

Before differential abundance analysis, we assessed the interaction between genetic line and WB scores. The Line × WB interaction was not significant for 88.04% of quantified lipids (FDR > 0.05; Fig. S3), supporting pooled downstream analyses with genetic line included as a covariate.

**Fig. 3:**
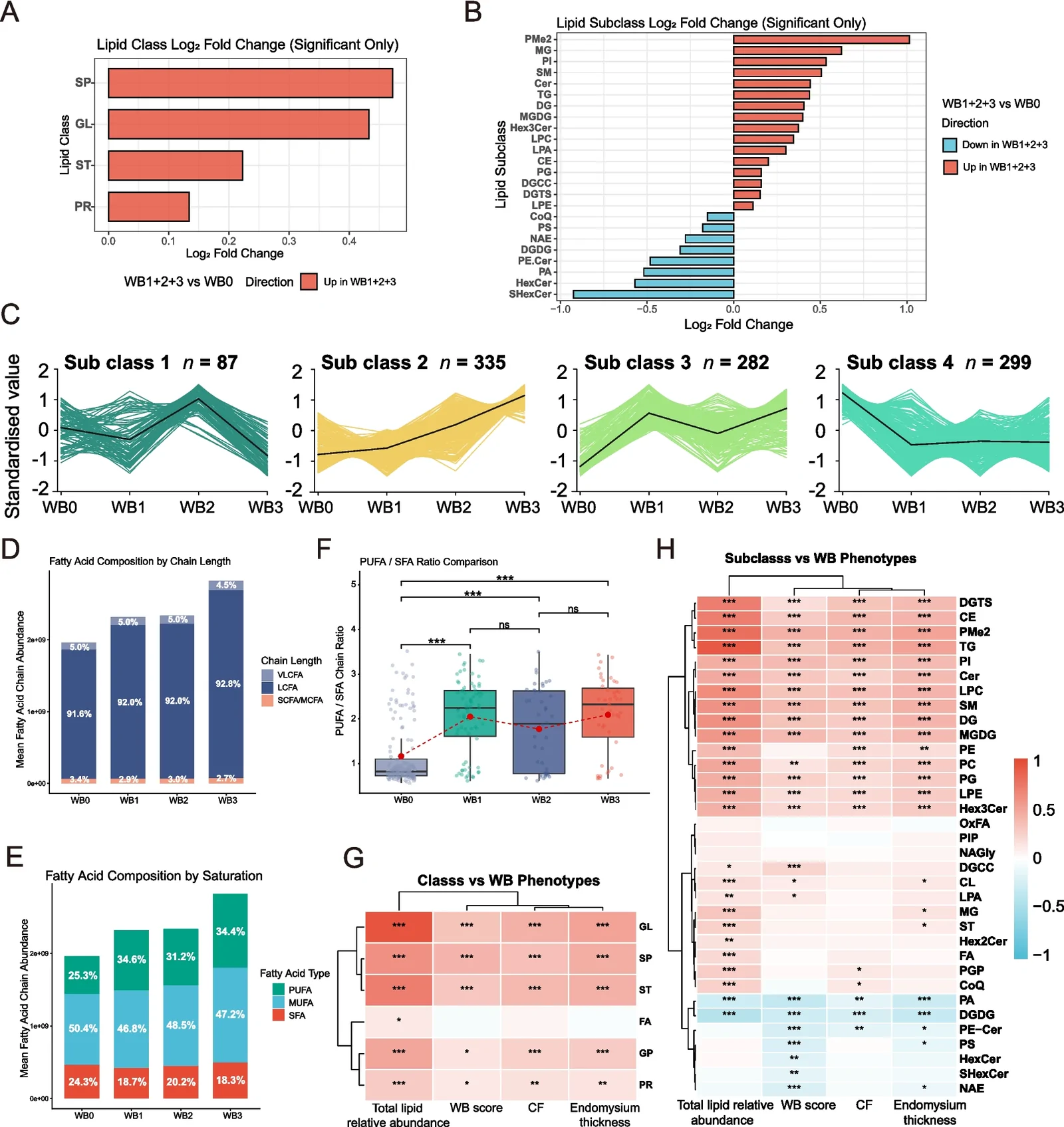
Lipidomic alterations associated with WB. **A** Bar plot showing the log₂ fold changes of significantly altered major lipid classes in the WB1 + 2 + 3 group compared with the WB0 group. WB1 + 2 + 3 denotes the pooled group of mild (WB1), moderate (WB2), and severe (WB3) WB samples. **B** Bar plot showing the log_2_FC of the 24 significantly altered lipid subclasses (*P* < 0.05) after removing unclassified lipid categories. **C** K-means clustering analysis revealing lipid species with dynamic abundance trends across WB scores. **D** Stacked bar plots representing the mean abundance and relative proportions of fatty acid chains categorized by chain length (SCFA/MCFA, LCFA, and VLCFA) across WB scores. **E** Stacked bar plots representing the mean lipid abundance of saturated fatty acids (SFA), monounsaturated fatty acids (MUFA), and polyunsaturated fatty acids (PUFA), along with their relative percentages. **F** Box plot of the PUFA/SFA ratio across WB scores. **G** and **H** Hierarchically clustered heatmaps of correlation coefficients between lipid major classes (**G**)/subclasses (**H**) and key phenotypic traits. The asterisks (*, **, ***) indicate statistical significance at *P* < 0.05, *P* < 0.01, and *P* < 0.001, respectively; ns indicates not significant (*P* ≥ 0.05)

WB was associated with extensive lipidomic remodeling. The summed lipid abundance differed between WB0 and WB1 + 2 + 3 samples for 24 lipid subclasses and four major lipid classes (Fig. 3A and B). K-means clustering further identified coordinated patterns of lipid abundance changes across WB scores (Fig. 3C).

Structural lipid composition also varied with WB scores. Fatty acid chain-length distributions were largely unchanged (Fig. 3D), whereas saturation profiles shifted toward a higher PUFA proportion and lower SFA proportion in WB samples (Fig. 3E), resulting in an increased PUFA/SFA ratio compared with WB0 (Fig. 3F).

Correlation analysis showed that lipid classes and subclasses exhibited stronger associations with total lipid abundance than with WB score or other traits, with glycerolipids (GLs), particularly TGs, showing the strongest positive correlations (Fig. 3G and H).

### Identification of WB-associated lipids via multi-strategy screening

**Fig. 4:**
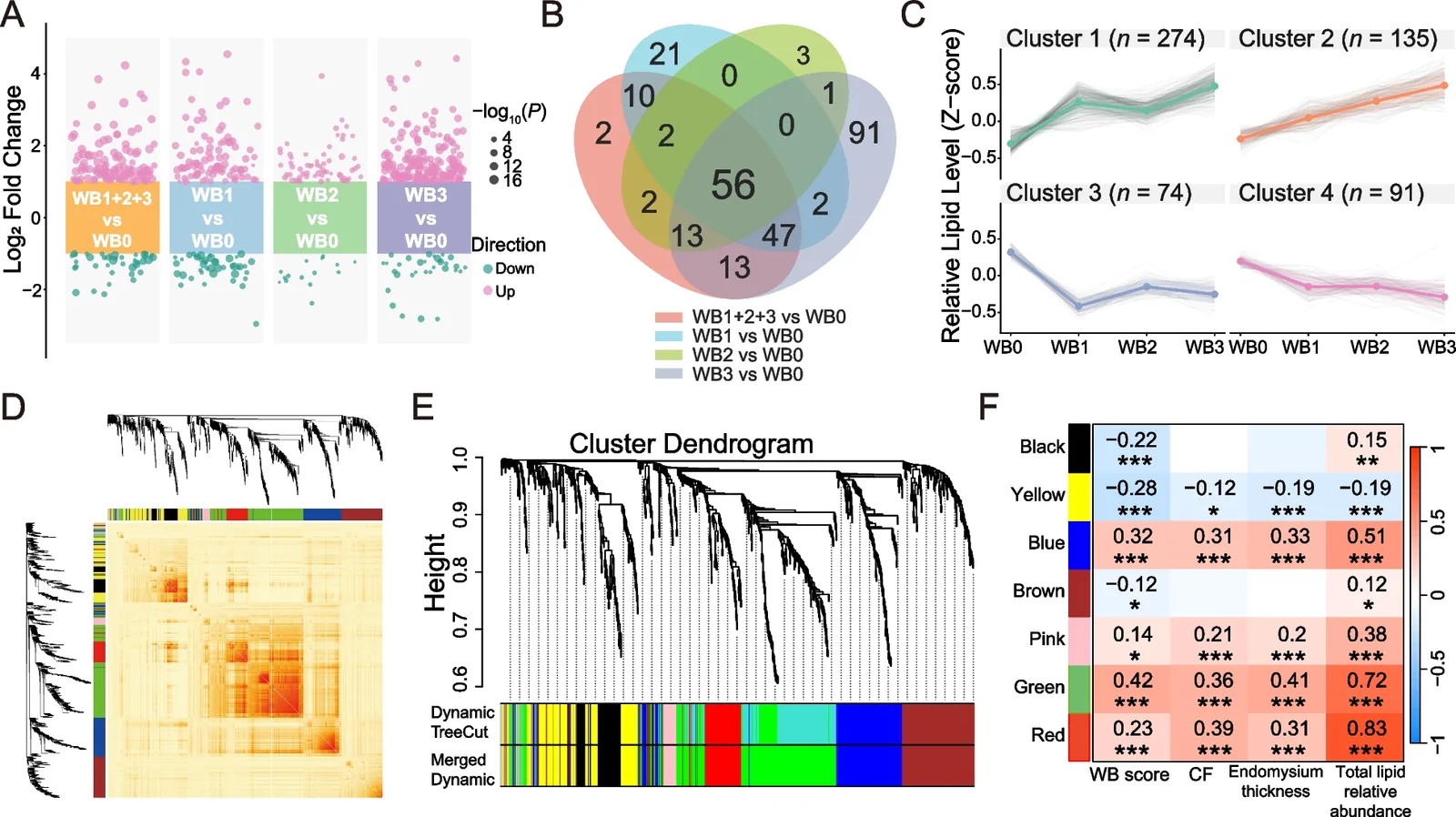
Identification of WB-associated lipids. **A** Volcano plots showing differential lipid abundance between WB0 and WB groups. **B** Venn diagram showing the overlap of differential lipids identified from the four comparisons in (**A**). **C** Clusters derived from trend analysis. **D** Heatmap of lipid–lipid similarity based on TOM, illustrating module-wise clustering patterns. **E** Hierarchical clustering dendrogram of lipid species based on TOM dissimilarity (1 − TOM). The upper panel shows clustering structure, and the lower panels indicate module assignments generated by dynamic tree cutting and module merging. **F** Module–trait correlation heatmap. Rows represent module eigengenes, and columns represent WB-associated phenotypic traits. Numbers indicate correlation coefficients, and asterisks (*, **, ***) indicate statistical significance at *P* < 0.05, *P* < 0.01, and *P* < 0.001, respectively

Differential abundance analysis was first performed between WB0 and different WB score groups. Volcano plots revealed extensive lipidomic alterations: 145 differential lipids in WB0 versus WB1 + 2 + 3 groups, 138 in WB0 versus WB1, 77 in WB0 versus WB2, and 223 in WB0 versus WB3 (Fig. 4A). These comparisons identified 56 lipids shared across all four comparisons (Fig. 4B, Table S1).

We next performed trend analysis to capture lipid abundance patterns across WB scores. A total of 574 lipids exhibited significant abundance patterns (FDR < 0.05) (Table S2), which were grouped into four major clusters (Fig. 4C). Cluster 2 (*n* = 135) showed a continuous increase, while Cluster 4 (*n* = 91) showed a continuous decrease with increasing WB severity. Clusters 1 (*n* = 274) and 3 (*n* = 74) displayed more complex patterns. Hierarchical clustering further showed consistent abundance patterns among these trend-associated lipids (Fig. S4).

We further applied WGCNA to identify co-expression modules linked to phenotypic traits. Hierarchical clustering confirmed no outliers among the 312 muscle samples (Fig. S5). Based on scale-free topology criteria, a soft-threshold power of 9 was selected (Fig. S6), and lipids were clustered into distinct modules (Fig. 4D and E). The module–trait association heatmap (Fig. 4F) highlighted three modules, Green, Red, and Blue, that were significantly and stably positively correlated with WB score, CF, endomysium thickness, and total lipid abundance. Within these modules, we observed strong correlations between module membership (MM) and gene significance (GS) (Fig. S7). Applying stringent thresholds (|MM| > 0.8 and |GS| > 0.2, *P* < 0.05), we identified 102 lipid species as hub lipids shared across all four phenotypes (Table S3). Notably, all 102 hub lipids were also included among the 574 trend-associated lipids, indicating strong agreement between the two complementary screening approaches.

Because the WB2 group contained fewer samples (*n* = 42) than the WB0 group (*n* = 152), we evaluated the statistical power of differential lipid analysis. Post-hoc power analysis indicated adequate power to detect moderate effect sizes (Fig. S8A). Consistent with this, although fewer differential lipids were detected in WB2, effect sizes remained highly correlated between WB2 and WB3 (Pearson *r* = 0.90, *P* < 0.001; Fig. S8B), suggesting that the reduced number of significant lipids in WB2 was primarily attributable to sample size.

### MR and mediation analysis of core lipids

**Fig. 5:**
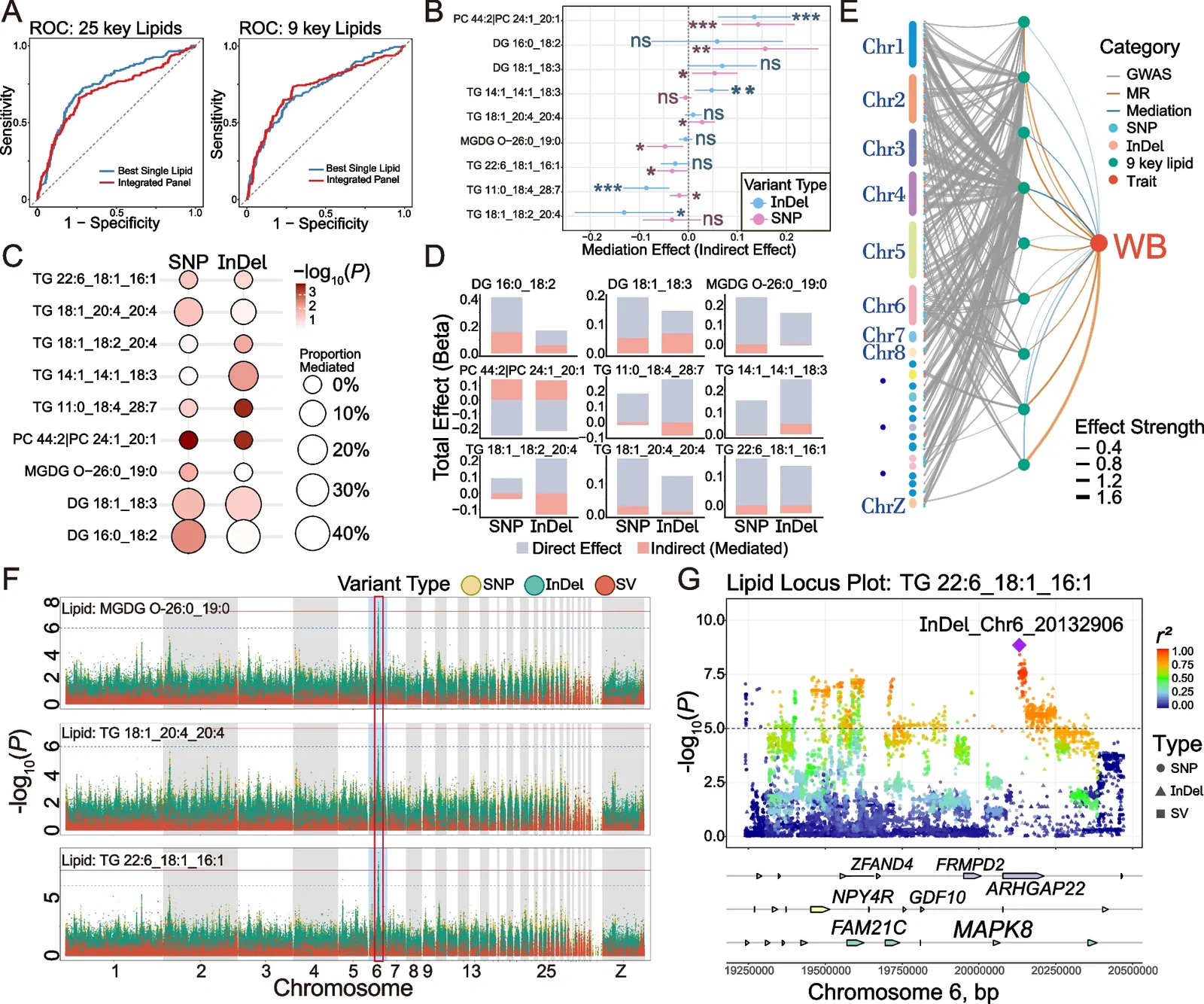
MR, mediation analysis and genomic loci mapping of core lipids. **A** Receiver operating characteristic curves comparing the diagnostic accuracy of the 25-lipid panel, the 9-lipid panel, and the single best-performing lipid marker. **B** Forest plots summarizing the effects estimated by MR for different genetic variants. **C** Bubble plots illustrate the significance and magnitude of mediation effects for SNPs and InDels. **D** Faceted plots for effect decomposition showing the direct and indirect contributions of genetic variants to WB phenotypes. **E** Integrative network diagram depicting the associated chain from genomic variants through lipids to the WB phenotype. **F** Manhattan plots for three representative lipid species showing significant association signals on chromosome 6. Each point represents a genetic variant (SNP, InDel, or SV). Genome-wide and suggestive significance thresholds are indicated. **G** Regional association locus plots for the candidate region on chromosome 6, accompanied by local LD structure and annotated genes within the interval. Asterisks (*, **, ***) indicate statistical significance at *P* < 0.05, *P* < 0.01, and *P* < 0.001, respectively; ns indicates not significant (*P* ≥ 0.05)

Integrating differential abundance analysis, trend analysis, and WGCNA identified a core panel of 25 candidate lipids (Table S4). Most showed progressively increased abundance across WB scores (Fig. S9). Collectively, these lipids achieved an AUC of 0.702 (95% CI: 0.643–0.761) for distinguishing WB from normal samples (Fig. 5A).

To evaluate whether genetically supported lipid variation was associated with WB, we applied a two-step framework integrating MR and mediation analysis using genetic variants as instrumental variables (Table S5). MR analysis identified eight SNP-based and six InDel-based lipid candidates for which the genetically predicted lipid levels were significantly associated with WB under the IVW model (Table S6). Subsequent mediation analyses retained seven SNP-derived and four InDel-derived candidates, and integration across both variant types yielded nine nonredundant high-priority lipids (Table S7, Table 3). Most prioritized lipids showed increased abundance across WB scores (Fig. S9).

**Table 3:** Identification and classification of candidate lipids

Lipid	Compound class	Lipid class	Ion mode	Lipid subclass	Major class
DG 18:1_18:3	DG	DG	Positive	DG	GL
DG 16:0_18:2	DG	DG	Positive	DG	GL
MGDG O-26:0_19:0	EtherMGDG	MGDG	Positive	MGDG	GL
PC 44:2|PC 24:1_20:1	PC	PC	Positive	PC	GP
TG 11:0_18:4_28:7	TG	TG	Positive	TG	GL
TG 14:1_14:1_18:3	TG	TG	Positive	TG	GL
TG 18:1_18:2_20:4	TG	TG	Positive	TG	GL
TG 18:1_20:4_20:4	TG	TG	Positive	TG	GL
TG 22:6_18:1_16:1	TG	TG	Positive	TG	GL

Mediation analysis further estimated the proportion of the genetic association with WB that was statistically mediated through lipid traits (Table S7). Among the prioritized candidates, TG 18:1_20:4_20:4 exhibited a significant indirect effect in the SNP-based mediation model (*P* = 0.034), explaining 15.62% of the total genetic association with WB. Supporting its prioritization, TG 18:1_20:4_20:4 also showed a positive MR association with WB score (IVW *P* = 0.010; Fig. 5B). The distribution of estimated mediation effects and statistical significance across candidate lipids is summarized in the bubble plot (Fig. 5C), while effect decomposition separated the total genetic association into direct and indirect components (Fig. 5D).

Sensitivity analyses, including leave-one-out analyses and alternative IV selection thresholds, indicated that MR estimates were consistent and were not substantially affected by individual instrumental variants (Tables S8 and S9).

Notably, the nine MR-prioritized lipids yielded a 10-fold cross-validation AUC of 0.729 (95% CI: 0.671–0.786), compared with 0.702 (95% CI: 0.643–0.761) for the initial 25-lipid panel, despite requiring fewer predictors (Fig. 5A). Although the nine-lipid panel showed a numerically higher AUC and higher sensitivity (Table S10), DeLong’s test indicated that the difference in AUC between the two models was not statistically significant (*P* = 0.1455), suggesting that model refinement improved parsimony while maintaining comparable predictive performance. The integrated network (Fig. 5E) summarizes the relationships among associated genetic loci, prioritized lipids, and WB phenotypic variation.

### Identification of a consensus genetic hotspot and candidate genes

To identify shared genetic regions associated with the nine prioritized lipids, we integrated the candidate intervals derived from mGWAS signals. Overlapping intervals revealed a consensus hotspot on chromosome 6 spanning 19.55–20.15 Mb. This hotspot was shared by three of the nine prioritized lipids—MGDG O-26:0_19:0, TG 22:6_18:1_16:1, and TG 18:1_20:4_20:4 (Fig. 5F; Tables S11–S13). Notably, InDel_Chr6_20132906 was the top InDel association for all three lipids and represented the strongest overall association signal for MGDG O-26:0_19:0 and TG 22:6_18:1_16:1.

Inspection of the regional association plot showed that the consensus hotspot overlapped a compact LD block containing seven annotated genes (Fig. 5G, Table 4). Transcriptomic analysis further prioritized five genes within the candidate interval that showed significant expression changes across WB scores (Fig. S10).

**Table 4:** Candidate genes within the chromosome 6 interval

Chromosome	Start	End	Gene ID	Gene name
6	19547702	19567861	ENSGALT00010051060	*ZFAND4*
6	19570384	19625587	ENSGALT00010051124	*FAM21C*
6	19642041	19643174	ENSGALT00010050336	*NPY4R*
6	19810279	19821960	ENSGALT00010050692	*GDF10*
6	19950431	20007144	ENSGALT00010049549	*FRMPD2*
6	20046995	20069065	ENSGALT00010049495	*MAPK8*
6	20078083	20212101	ENSGALT00010049567	*ARHGAP22*

To evaluate whether this locus was associated with broader lipidomic variation, we further performed mGWAS of lipid class and subclass abundance traits. Multiple traits, including CE, ST, GL, TG, and PMe2, displayed association peaks within the same chromosome 6 interval, and LD clumping identified independent lead variants in this region (Fig. S11; Table S14). Moreover, the nine prioritized lipids were positively correlated with these lipid traits (Fig. S12). These findings support the chromosome 6 interval as a candidate genetic region associated with lipid variation related to WB.

### Independent validation of lipid biomarkers and multi-omics candidates

**Fig. 6:**
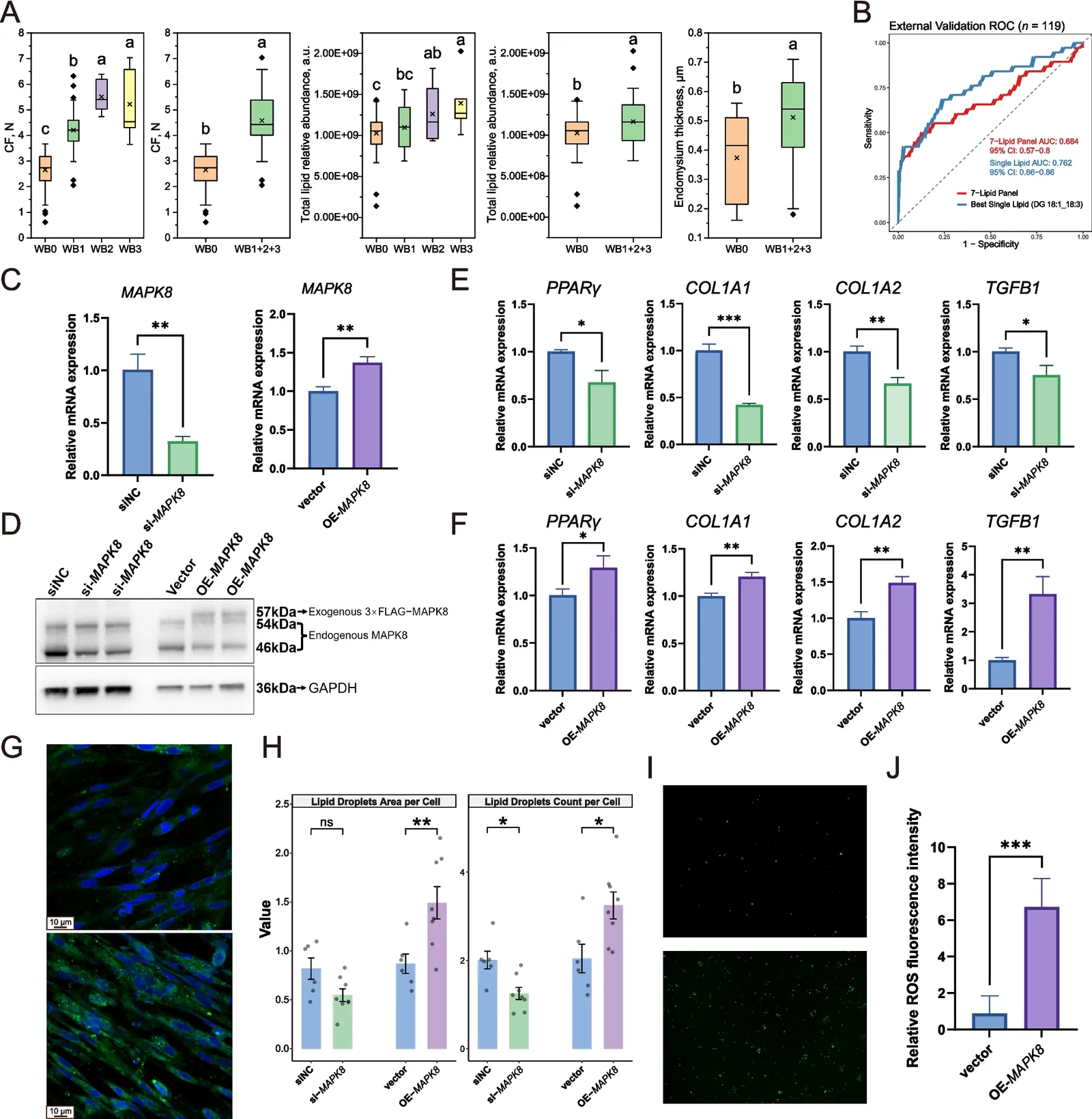
Independent validation of the lipid biomarker and functional characterization of *MAPK8* in chicken primary myoblasts. **A** Phenotypic differences between WB0 and WB-affected birds in the independent cohort. *n* = 119 for CF and total lipid relative abundance, and *n* = 27 for endomysium thickness. **B** ROC analysis of the seven-lipid biomarker panel in the independent validation cohort using the predefined cutoff from the discovery cohort. **C** Relative *MAPK8* mRNA expression following siRNA-mediated knockdown (left) and plasmid-mediated overexpression (right). **D** Western blot analysis confirming *MAPK8* knockdown and overexpression. The MAPK8 antibody detected two endogenous bands (~46 and ~54 kDa), while the upper band (~57 kDa) corresponds to the exogenous 3 × FLAG-tagged MAPK8 fusion protein. **E** Relative mRNA expression of *PPARγ*, *COL1A1*, *COL1A2*, and *TGFB1* following *MAPK8* knockdown. **F** Relative mRNA expression of *PPARγ*, *COL1A1*, *COL1A2*, and *TGFB1* following *MAPK8* overexpression. **G** Representative fluorescence images of lipid droplets stained with BODIPY (green) and nuclei stained with DAPI (blue) in *MAPK8* knockdown and overexpression experiments. Scale bar = 10 μm. **H** Quantification of lipid droplet area per cell and lipid droplet count per cell following *MAPK8* knockdown and overexpression. **I** Representative images of intracellular ROS detected using DCFH-DA fluorescence staining in the vector control and *MAPK8* overexpression groups (4 ×). **J** Quantification of intracellular ROS fluorescence intensity. Data are presented as mean ± SD. Different lowercase letters indicate significant differences between groups (*P* < 0.05). ns indicates no significant difference, while *, **, and *** indicate *P* < 0.05, *P* < 0.01, and *P* < 0.001, respectively

To evaluate the robustness of the identified lipid biomarkers and candidate genes, an independent cohort comprising 119 broilers was analyzed. Consistent with the discovery cohort, WB samples exhibited significantly increased CF, endomysium thickness, and total lipid abundance (Fig. 6A), confirming the pathological consistency of the validation cohort.

Of the nine prioritized lipid biomarkers identified in the discovery cohort, seven were detected in the independent cohort. All seven showed significantly higher abundance in WB samples, with effect directions consistent between the discovery and validation cohorts (Fig. S13; Table S15). These seven lipids were subsequently used to construct an externally validated lipid panel, which achieved an AUC of 0.684 (95% CI: 0.567–0.801) using the predefined cutoff from the discovery cohort (Fig. 6B; Table S16). Individual lipids showed comparable diagnostic performance (Table S17), and no significant difference was detected between the panel and the best-performing single lipid (DeLong's test, *P* = 0.075).

Transcriptomic validation further supported significantly increased expression of *MAPK8* and *NPY4R* in WB samples (Fig. S14). *MAPK8* was subsequently selected for functional characterization in chicken primary myoblasts.

### Functional characterization of *MAPK8* in chicken primary myoblasts

To investigate the potential involvement of *MAPK8* in WB-associated cellular phenotypes, *MAPK8* was knocked down or overexpressed in primary chicken myoblasts. qPCR and Western blot analyses confirmed efficient knockdown and overexpression (Fig. 6C, D; Fig. S15). Modulating *MAPK8* expression was accompanied by changes in the expression of genes involved in lipid metabolism and extracellular matrix remodeling, including *PPARγ*, *COL1A1*, *COL1A2*, and *TGFB1* (Fig. 6E and F).

In addition, BODIPY neutral lipid staining (Fig. 6G and H) and ROS fluorescence staining (Fig. 6I and J) revealed a consistent pattern, with fluorescence intensities being highest in the *MAPK8* overexpression group, and lowest in the control group or the knockdown group. Together, these findings indicate that increased *MAPK8* expression is associated with enhanced intracellular oxidative stress, lipid accumulation, and profibrotic gene expression in chicken primary myoblasts, supporting its potential involvement in cellular processes relevant to WB.

## Discussion

WB is a multifactorial, growth-associated myopathy characterized by progressive fibrosis and impaired muscle quality [41]. In the present study, WB-affected muscles exhibited increased collagen deposition, expansion of the endomysial space, and elevated intramuscular lipid abundance, consistent with the established pathological features of fibrosis and adipose infiltration [42]. Histological examination further revealed disorganized type I and III collagen deposition and pronounced connective tissue remodeling, supporting the hypothesis that excessive extracellular matrix accumulation contributes to the increased hardness of WB muscle [43, 44]. Together with increased total lipid abundance, these findings indicate that structural remodeling and lipid accumulation are closely associated with WB [45–47].

Lipidomic profiling further revealed extensive remodeling of muscle lipid metabolism, with TGs and PCs representing the predominant lipid classes enriched in WB. Previous studies have similarly reported early activation of fatty acid uptake and lipid storage pathways in birds that subsequently develop WB, suggesting that altered lipid metabolism is an early feature of WB [48–50]. Excessive intramyocellular TG accumulation has been considered a hallmark of lipotoxicity and metabolic dysfunction [51], whereas elevated PCs may reflect increased membrane turnover and compensatory repair following myofiber injury [52, 53]. In addition, the increased PUFA/SFA ratio observed in WB suggests active remodeling of membrane lipid composition rather than simple lipid accumulation.

Lipid accumulation within non-adipose tissues is frequently accompanied by oxidative stress, endoplasmic reticulum stress, and inflammatory responses [53–55]. These processes have also been extensively reported in WB muscle and are considered important contributors to myofiber degeneration and subsequent fibrotic remodeling [56]. Under the rapid growth and high metabolic demand characteristic of modern broilers, excessive nutrient influx may exceed the metabolic capacity of skeletal muscle, promoting intracellular lipid accumulation and metabolic stress [57, 58]. Persistent cellular stress may subsequently promote extracellular matrix deposition and connective tissue expansion, potentially contributing to the increased hardness characteristic of WB [59]. Consistent with these observations, our results demonstrate that abnormal lipid remodeling is closely associated with oxidative stress and fibrotic remodeling across different WB severity scores. This provides a biological rationale for prioritizing lipid species that may better reflect metabolic alterations associated with WB.

Conventional differential lipidomics identifies lipids associated with WB severity but cannot distinguish genetically supported metabolic traits from secondary metabolic alterations. By integrating MR and mediation analyses, we prioritized nine lipid species showing evidence of genetic association with WB. Compared with the initial 25-lipid panel, the refined panel retained comparable discriminatory performance while improving model stability, suggesting that genetically prioritized lipid traits may better reflect biologically relevant metabolic variation associated with WB. However, as this study is observational and cross-sectional, these findings should be interpreted as genetically supported evidence rather than definitive proof of causality.

Beyond biomarker prioritization, several of the genetically prioritized lipids also provided insights into the biological processes underlying WB. Among the prioritized lipids, TG 18:1_18:2_20:4 was positively correlated with the expression of *ME1* and *INSIG1*, two genes involved in de novo lipogenesis and sterol homeostasis [60]. Rather than representing passive metabolic by-products, highly unsaturated TG species may reflect coordinated regulation of lipid synthesis, storage, and membrane remodeling, consistent with previous studies linking complex TG species to adipogenic differentiation and muscle quality traits [61]. These observations support the concept that specific TG species may serve as metabolically informative indicators of lipid dysregulation in WB.

An additional strength of this study is that three prioritized lipids converged on a shared genomic interval on chromosome 6, suggesting a candidate regulatory interval rather than independent genetic signals. The convergence of multiple lipid traits within the same genomic interval suggests coordinated genetic regulation of lipid metabolism rather than isolated effects on individual metabolites. The overlap of this interval with previously reported WB-associated loci further supports its potential biological relevance, such as SNP_Chr6_19640631 [62]. Instead of implicating a single causal gene, this locus is more likely to represent a regulatory region influencing multiple lipid-associated traits, consistent with the polygenic architecture of complex metabolic traits. Integrating mGWAS with transcriptomic profiling enabled further prioritization of genes showing expression changes concordant with WB scores, thereby substantially narrowing the list of biologically plausible candidates.

Several candidate genes within this locus have reported functions related to membrane trafficking, protein quality control, cytoskeletal organization, and metabolic regulation. For example, *FAM21C* participates in WASH complex-mediated endosomal trafficking and has been implicated in lipid droplet dynamics [63–66], whereas *ZFAND4* is associated with cellular stress responses [67, 68]. *FRMPD2* and *NPY4R* are linked to cytoskeletal organization and metabolic homeostasis, respectively [69–71]. Although these biological functions are consistent with the pathological features of WB, their specific roles in WB development remain speculative and require direct functional validation.

Among the prioritized candidates, *MAPK8* was supported by both transcriptomic and functional analyses. Previous transcriptomic and co-expression studies in chickens have identified *MAPK8* as a candidate regulator of intramuscular fat deposition through pathways involved in lipid synthesis and energy metabolism, supporting a potential role for *MAPK8* in muscle lipid homeostasis [72]. Consistent with these observations, *MAPK8* expression increased predominantly in severe WB, and its overexpression in primary chicken myoblasts promoted lipid accumulation, oxidative stress, and the expression of fibrosis-related genes, whereas *MAPK8* knockdown produced the opposite effects.

These findings are consistent with the reported role of JNK signaling in coordinating lipotoxic stress, mitochondrial dysfunction, oxidative stress, and inflammatory responses in skeletal muscle [73, 74]. *MAPK8* has also been implicated in regulating myoblast proliferation, differentiation, and apoptosis through the JNK pathway [75], suggesting that stress-responsive MAPK signaling may influence both muscle development and pathological remodeling. Recent reviews suggest that persistent lipid overload activates stress-responsive MAPK/JNK signaling, which is closely associated with increased ROS production, mitochondrial dysfunction, and extracellular matrix remodeling [76–78]. Consistent with these observations, our functional experiments suggest that *MAPK8* may participate in stress responses and fibrotic remodeling associated with WB, although its precise role within the regulatory network requires further investigation in vivo.

Beyond providing mechanistic insights, these genetically prioritized lipid traits may also have translational value. The lipid species identified in this study represent metabolically informative traits with potential heritability. Previous studies have demonstrated that metabolites with moderate genetic *h*^2^ can serve as effective biomarkers for selection in poultry [79]. If validated, WB-associated lipid metabolites may be useful for incorporation into breeding and nutritional strategies aimed at reducing WB incidence.

This study has several limitations. First, lipidomic and phenotypic measurements were obtained at a single developmental stage, preventing direct assessment of the temporal sequence between lipid remodeling and WB. Consequently, although MR and mediation analyses provide genetically informed evidence supporting associations between prioritized lipid traits and WB, they cannot by themselves establish causal relationships. Second, the relatively smaller sample sizes in the WB2 and WB3 groups may have reduced statistical power to detect subtle metabolic alterations. Nevertheless, the high concordance of lipid effect sizes across WB scores suggests that the overall metabolic remodeling pattern was biologically consistent. Future longitudinal studies, larger validation cohorts, and additional functional experiments will be necessary to further refine the regulatory mechanisms identified in this study.

Despite these limitations, integrating lipidomics with genetic association analyses enabled the prioritization of biologically informative lipid traits and substantially narrowed the search space for candidate regulatory genes. This integrative strategy provides a general framework for linking metabolic phenotypes with genetic variation and downstream functional prioritization in complex traits.

## Conclusion

In conclusion, WB-affected muscles exhibited increased muscle hardness, increased endomysial connective tissue deposition, and elevated intramuscular lipid abundance. Integrative analyses revealed extensive lipid metabolic alterations associated with WB and identified candidate lipid markers and genetic factors potentially involved in this complex myopathy. Furthermore, functional validation supported a potential role of *MAPK8* in WB-associated lipid accumulation, oxidative stress, and fibrotic remodeling in chicken muscle. Collectively, this study provides new insights into the molecular mechanisms underlying WB and establishes a foundation for future genetic and functional investigations aimed at improving our understanding and management of this economically important myopathy.

## Supplementary Information

Additional file 1: Table S1. Differentially abundant lipids identified by limma analysis. Table S2. Lipids with significant abundance trends across WB scores. Table S3. Hub lipids identified from significant WGCNA modules. Table S4. Consensus lipids identified across differential analysis, trend analysis, and WGCNA. Table S5. Detailed statistics of IVs used in MR analysis. Table S6. Significant MR results for candidate lipids associated with WB. Table S7. Significant indirect effects identified by two-step MR mediation analysis. Table S8. Leave-one-out sensitivity analysis for MR estimates. Table S9. Sensitivity analysis using different IV selection thresholds. Table S10. Diagnostic performance of lipid panels and the best-performing individual lipids for WB classification. Table S11. Significant mGWAS loci. Table S12. Suggestive mGWAS loci. Table S13. Candidate genomic regions defined by LD clumping. Table S14. Independent lead variants identified within the chromosome 6 candidate interval by LD clumping. Table S15. External validation of differential abundance and effect direction for the seven replicated lipid biomarkers in the independent validation cohort. Table S16. Performance metrics of the 7-lipid panel in the independent validation cohort. Table S17. ROC performance of individual lipid markers in the independent validation cohort.

Additional file 2: Fig. S1. Phenotypic distribution and genomic differentiation between the two broiler lines. Fig. S2. Illustration of endomysium thickness measurement. Fig. S3. Volcano plot of Line × WB interaction effects across 1,003 lipid species. Fig. S4. Hierarchical clustering of trend-associated lipids. Fig. S5. Global sample clustering. Fig. S6. WGCNA network topology analysis. Fig. S7. MM versus GS scatter plots. Fig. S8. Evaluation of statistical power and consistency of lipid alterations across WB scores. Fig. S9. Abundance of 25 candidate lipids across WB scores. Fig. S10. Differential expression of candidate genes. Fig. S11. Manhattan plots of lipid class and subclass abundance traits highlighting the chromosome 6 candidate locus. Fig. S12. Correlation matrix between the nine prioritized lipids and lipid class/subclass traits associated with the chromosome 6 candidate locus. Fig. S13. Validation of seven lipid biomarkers in the independent cohort. Fig. S14. Expression validation of candidate genes in the independent cohort. Fig. S15. Original uncropped Western blot images for MAPK8 and GAPDH.

## Data Availability

The datasets used during the current study are available from the corresponding author on reasonable request.

## Abbreviations

AUC: Area under the receiver operating characteristic curve
CF: Compression force
DGs: Diacylglycerols
FAs: Fatty acyls
FBS: Fetal bovine serum
GLs: Glycerolipids
GPs: Glycerophospholipids
*h*^2^: Heritability
HE: Hematoxylin–eosin
InDels: Insertions/deletions
IVs: Instrumental variables
IVW: Inverse variance weighted
LD: Linkage disequilibrium
MGDG: Monogalactosyldiacylglycerol
mGWAS: Metabolome genome‐wide association study
MR: Mendelian randomization
MUFAs: Monounsaturated fatty acids
PCs: Phosphatidylcholines
PCA: Principal component analysis
PEs: Phosphatidylethanolamines
PRs: Prenol lipids
PSs: Phosphatidylserines
PUFAs: Polyunsaturated fatty acids
QC: Quality control
ROC: Receiver operating characteristic
SFA: Saturated fatty acids
SLs: Sphingolipids
SNPs: Single nucleotide polymorphisms
STs: Sterol lipids
SVs: Structural variants
TGs: Triacylglycerols
WB: Wooden breast
WGCNA: Weighted gene co-expression network analysis
