# Supplementary material for: Integrative lipidomic and genomic analysis identifies key lipids for wooden breast in chickens
Source: J Anim Sci Biotechnol. 2026 Nov 3;17:198. doi: 10.1186/s40104-026-01517-5 (PMC13729490; doi:10.1186/s40104-026-01517-5)
Supplement: Supplementary file 1 [file 40104_2026_1517_MOESM2_ESM.pdf]

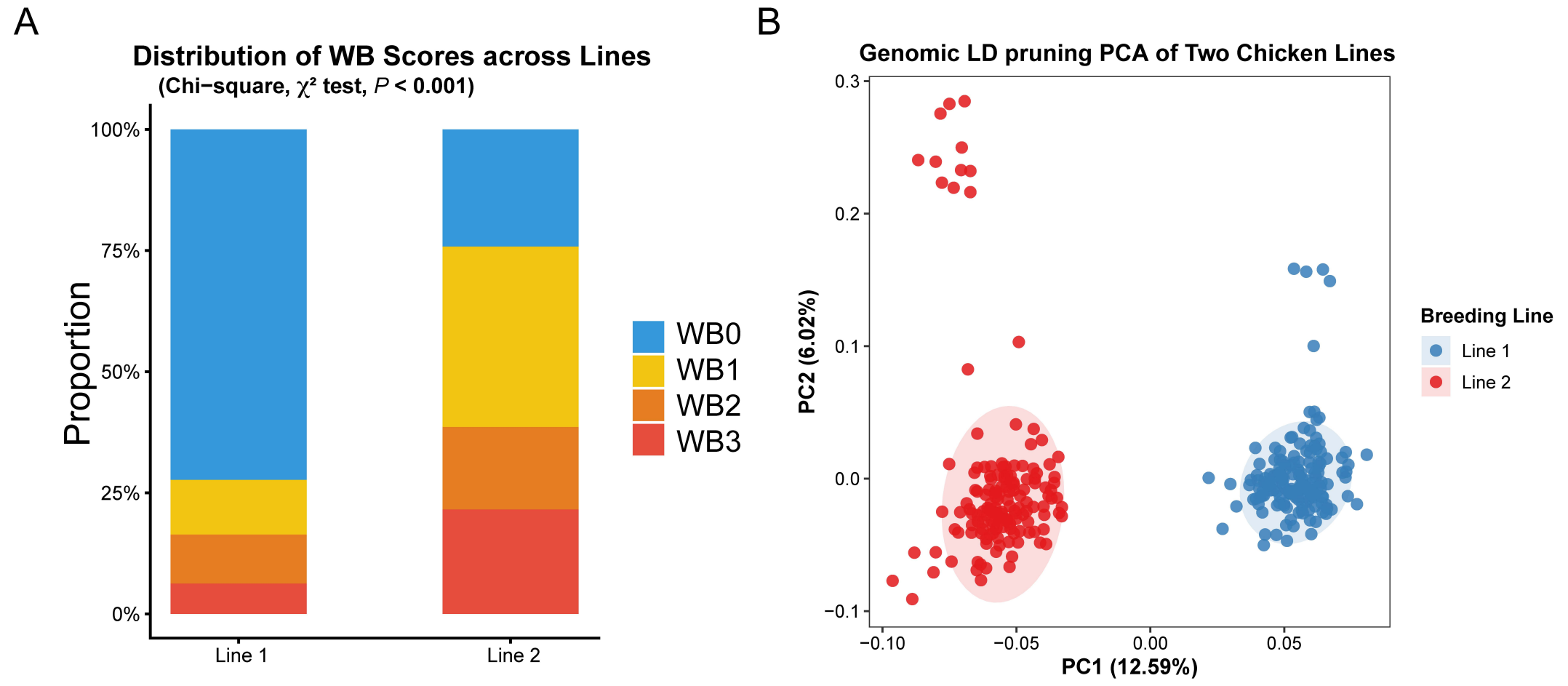

Figure S1. Phenotypic distribution and genomic differentiation between the two commercial broiler lines. (A) Distribution of WB scores across lines. (B) Principal component analysis based on LD-pruned genome-wide SNP markers revealed clear separation between the two commercial breeding lines.

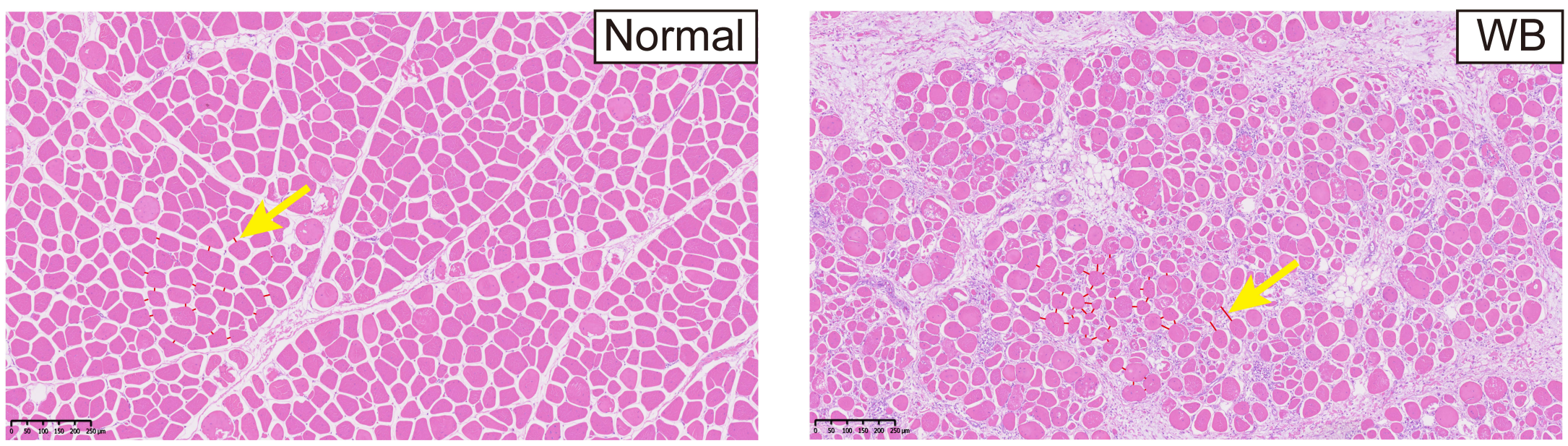

Figure S2. Illustration of endomysium thickness measurement. Yellow arrows indicate red line segments representing endomysium thickness.

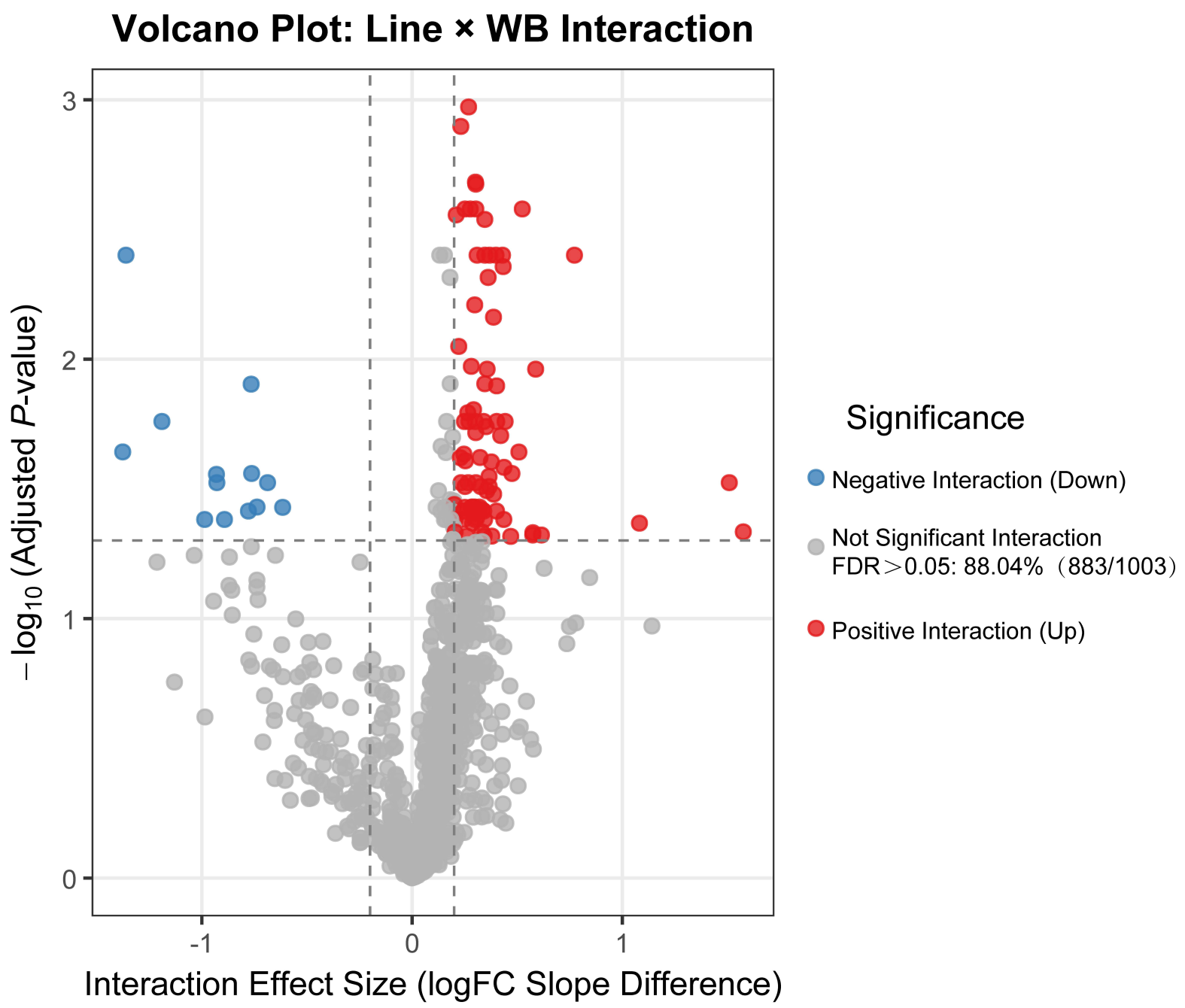

Figure S3. Volcano plot of Line × WB interaction effects across 1,003 lipid species.

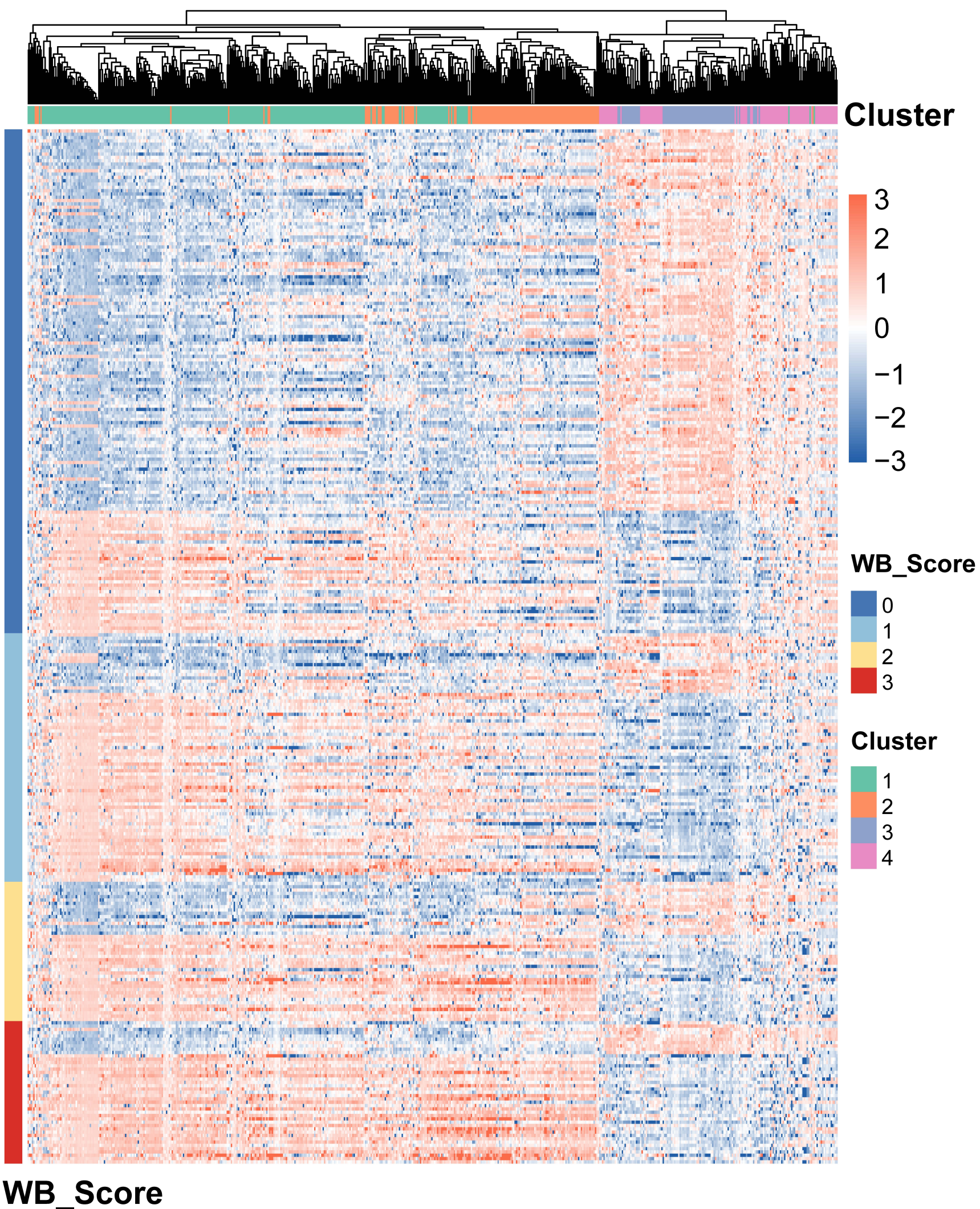

Figure S4. Hierarchical clustering of trend-associated lipids.

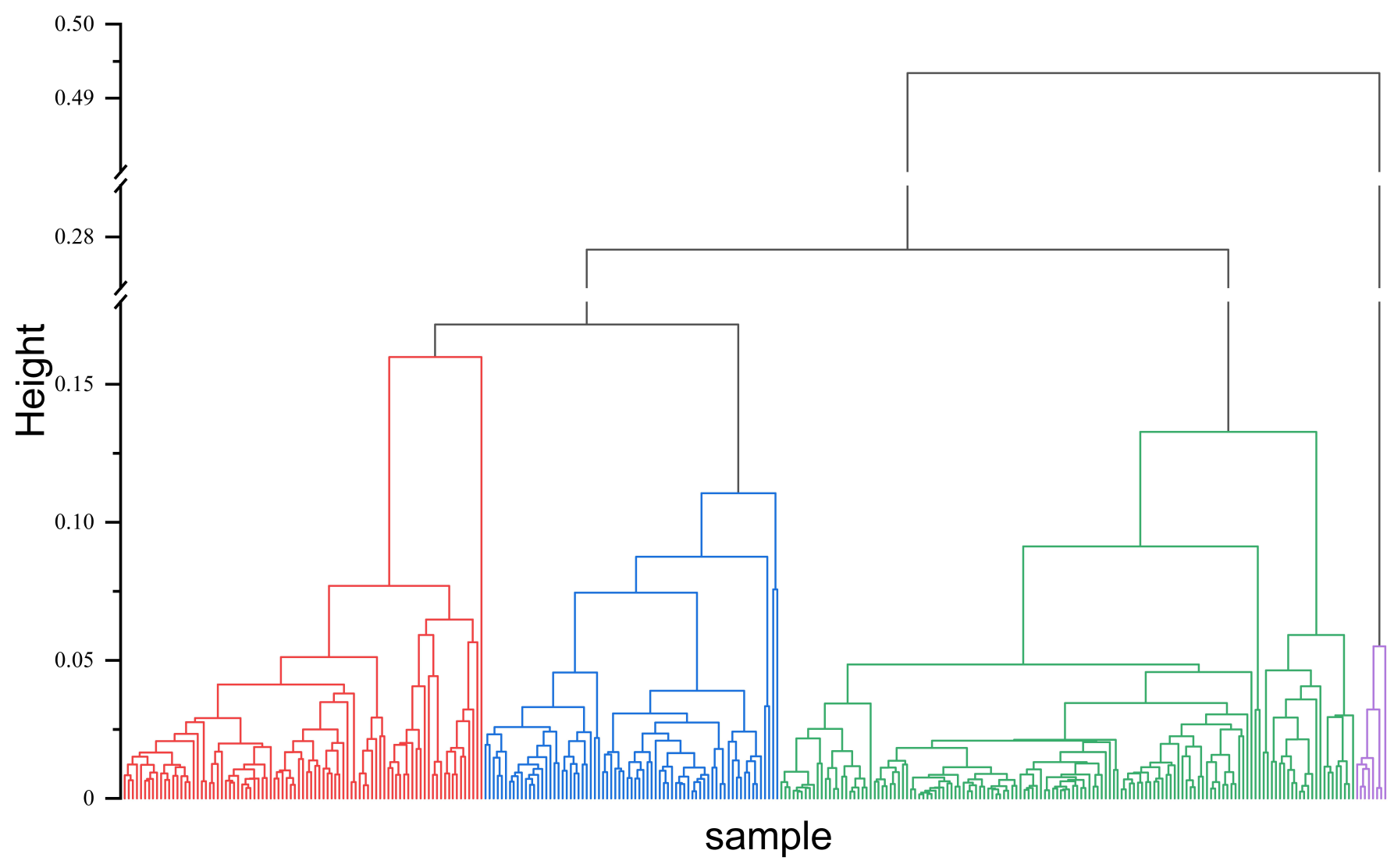

Figure S5. Global sample clustering. Hierarchical clustering of all 312 samples.

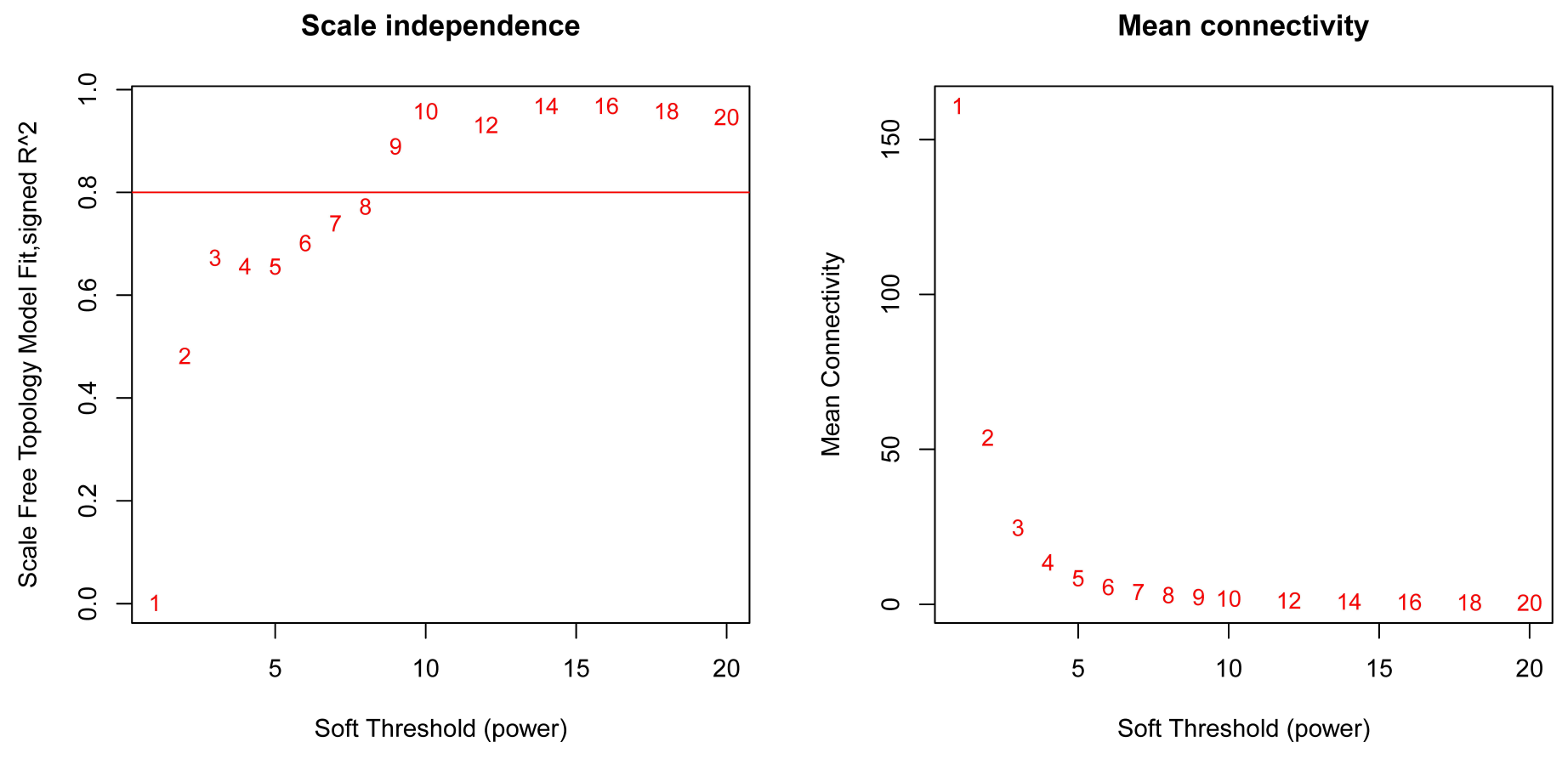

Figure S6. WGCNA network topology analysis. Plots of the scale-free fit index and mean connectivity across various soft-thresholding powers.

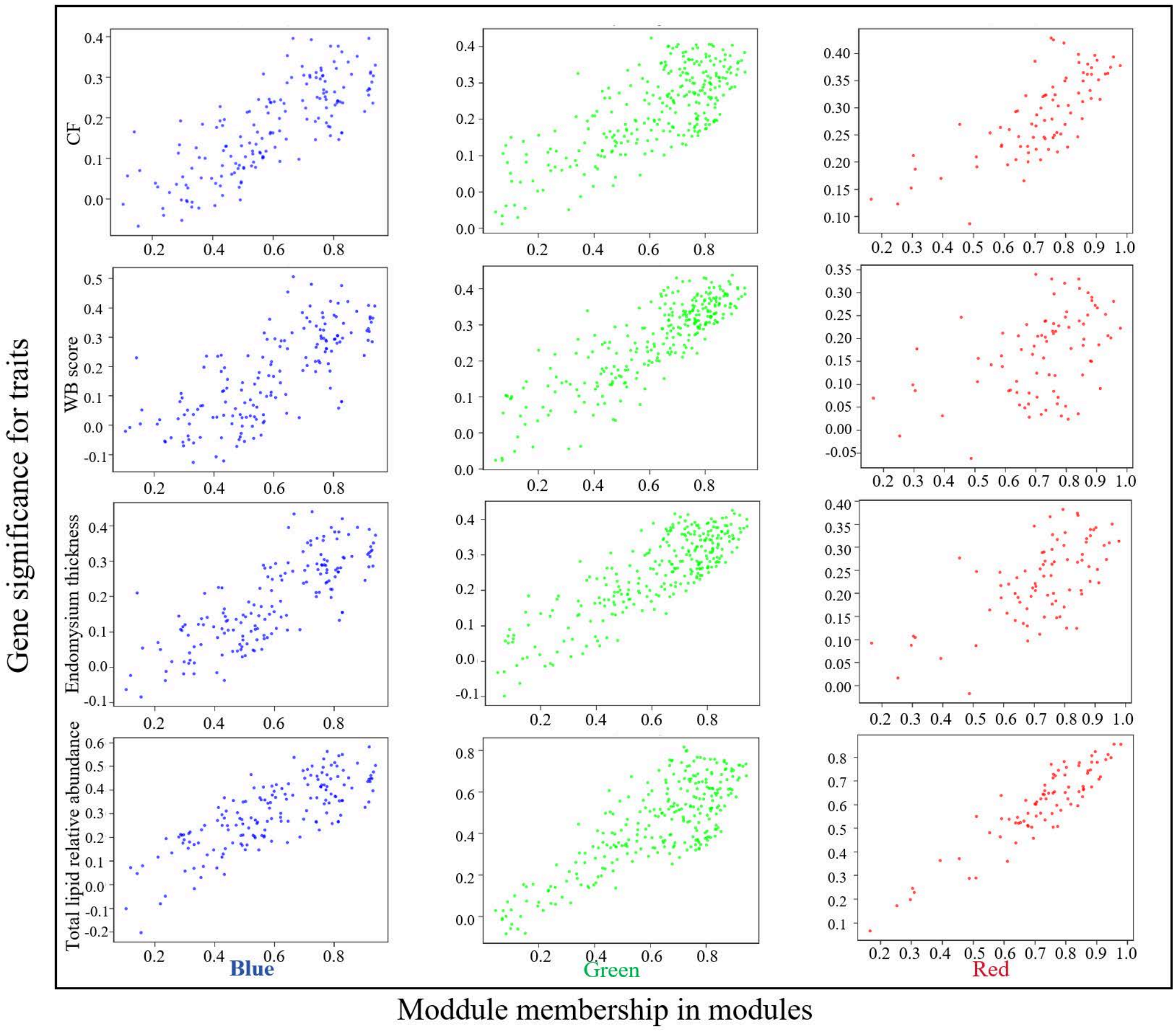

Figure S7. MM versus GS scatter plots. Correlations between MM and GS for traits within the Green, Red, and Blue modules.

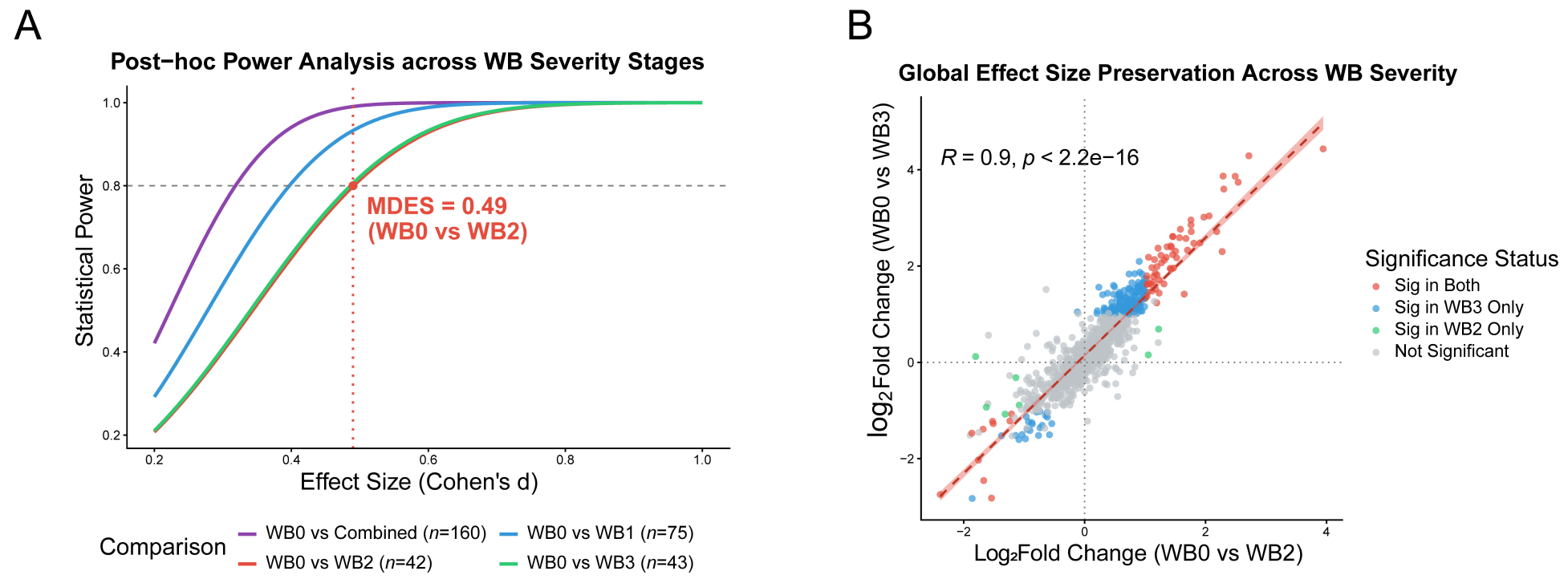

Figure S8. Evaluation of statistical power and consistency of lipid alterations across WB scores. (A) Power curve. (B) Correlation of lipid effect sizes between WB2 and WB3.

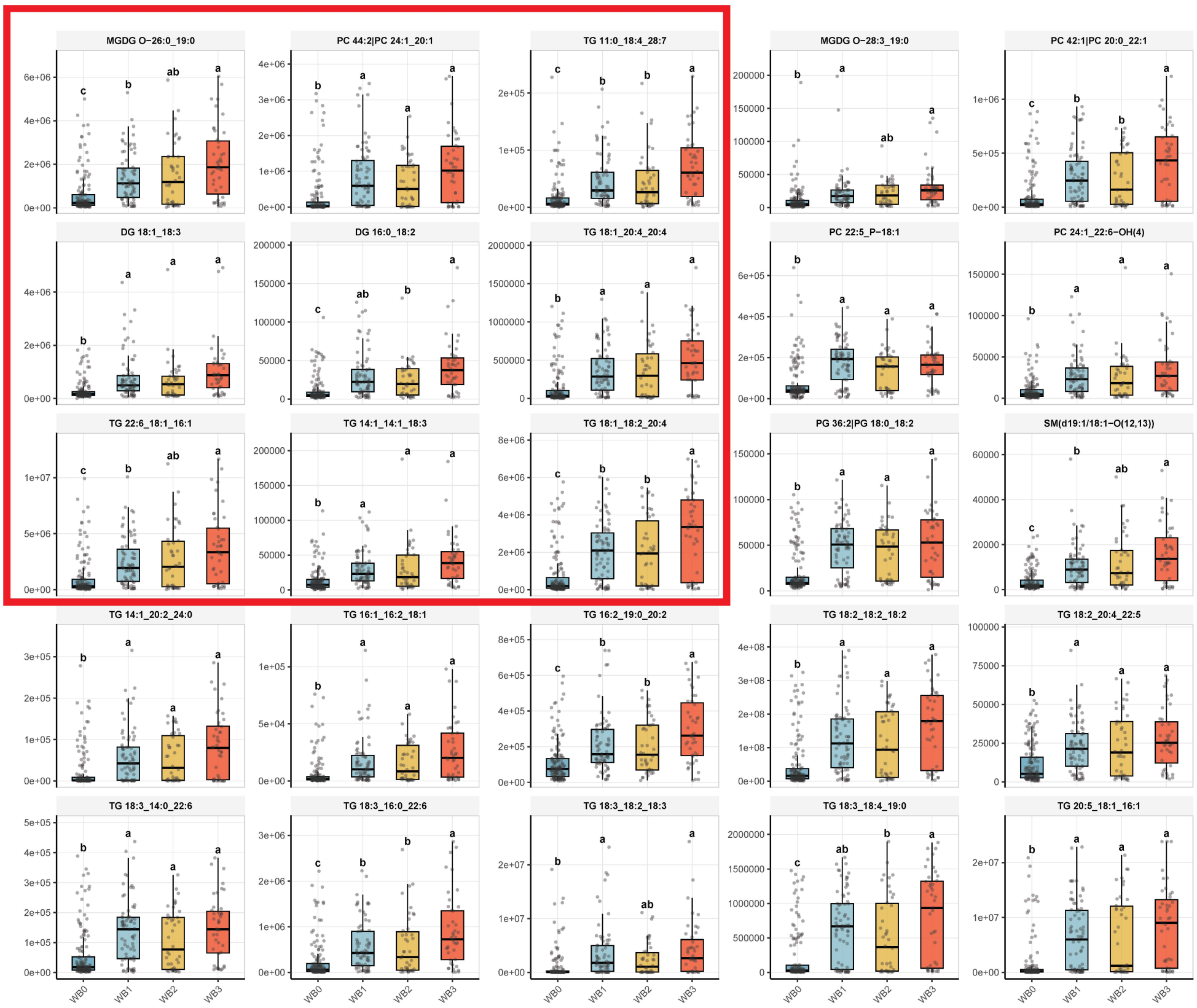

Figure S9. Abundance of 25 candidate lipids across WB scores. Box plots show the distribution of each lipid; the 9 prioritized lipids are highlighted with red frames. Different lowercase letters indicate significant differences among WB scores ($P < 0.05$).

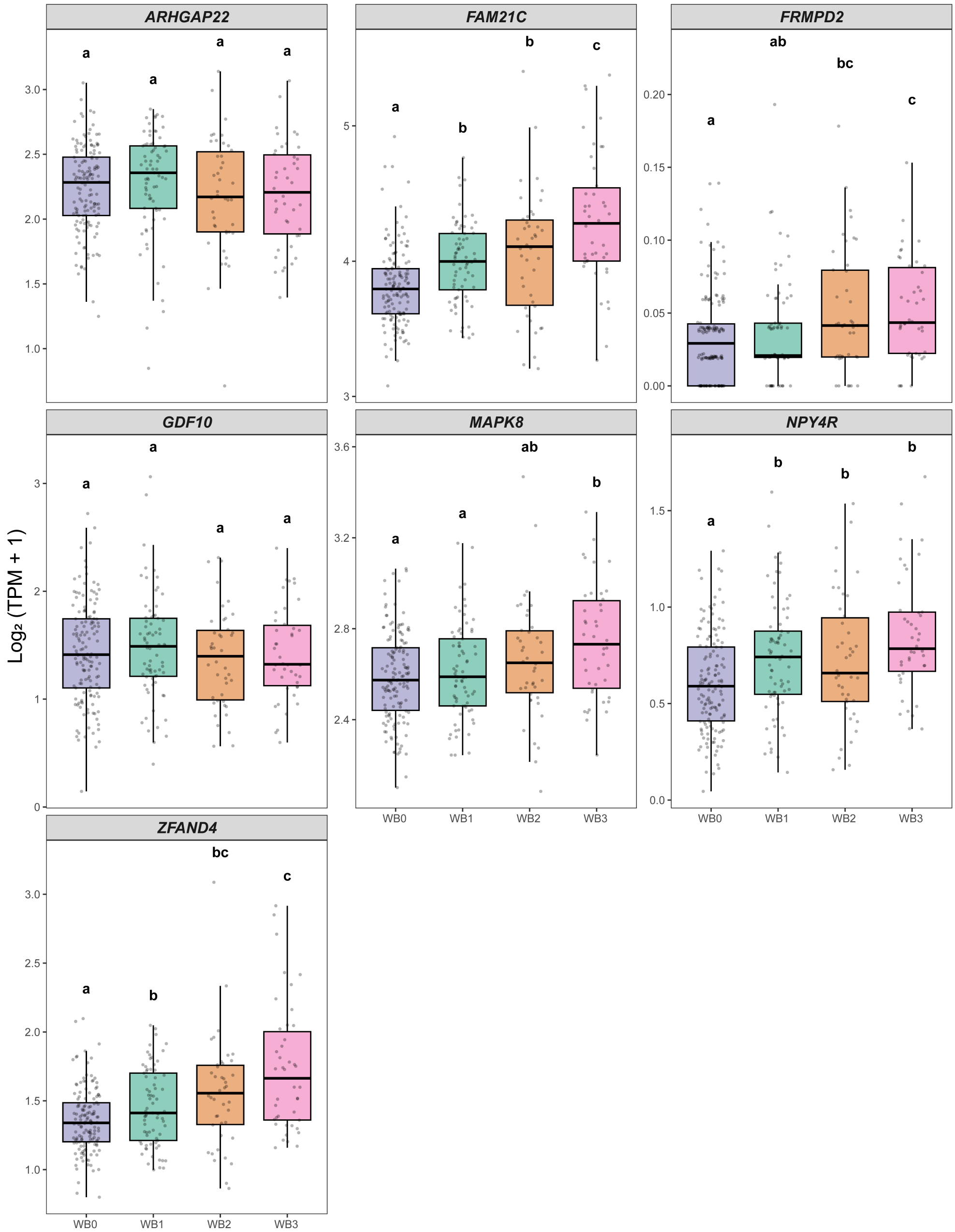

Figure S10. Differential expression of candidate genes. Box plots comparing mRNA expression levels (TPM) of the seven annotated genes within the chromosome 6 interval between WB0 and WB-affected groups. Different lowercase letters indicate significant differences ($P < 0.05$).

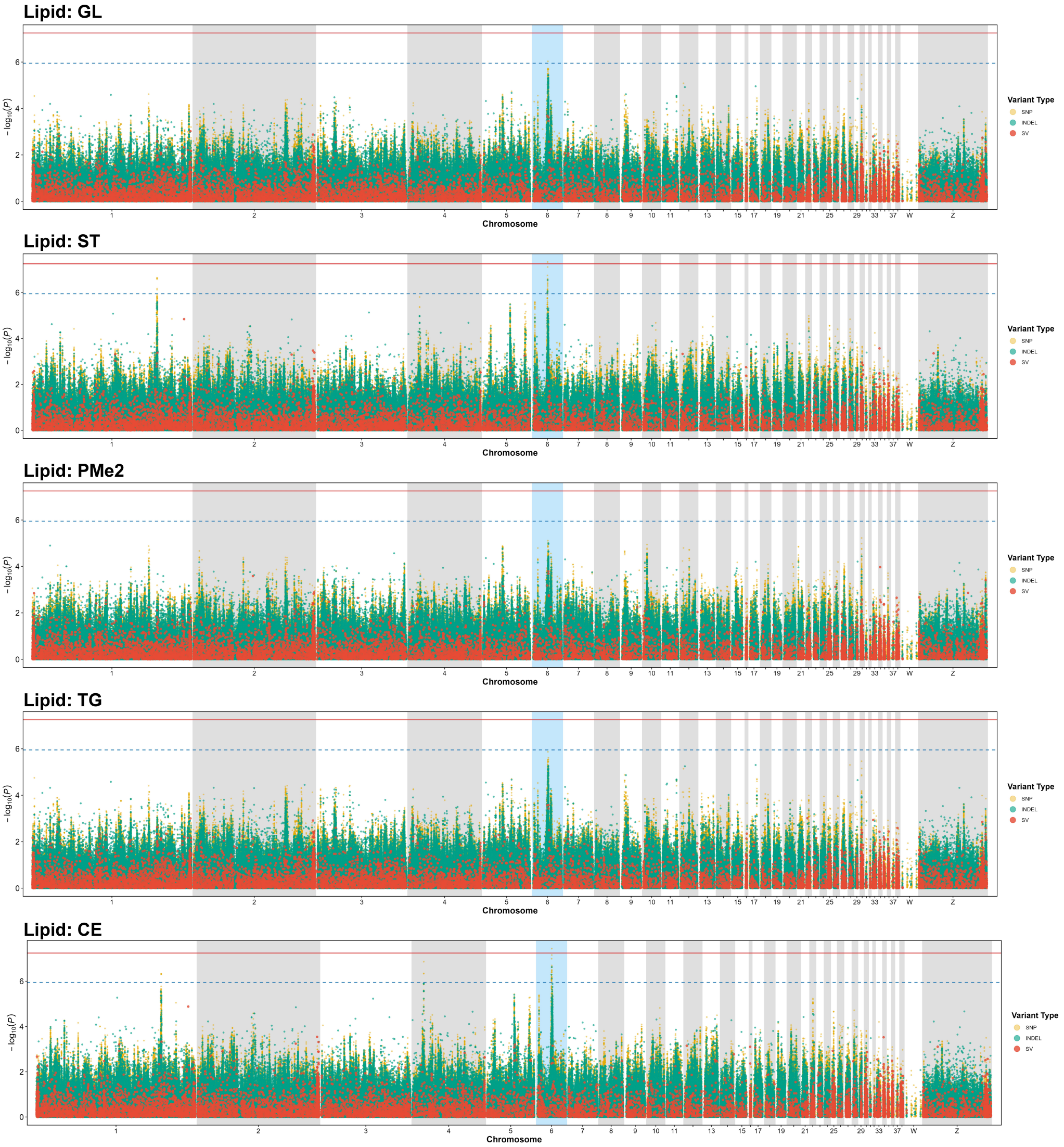

Figure S11. Manhattan plots of lipid class and subclass abundance traits highlighting the chromosome 6 candidate locus.

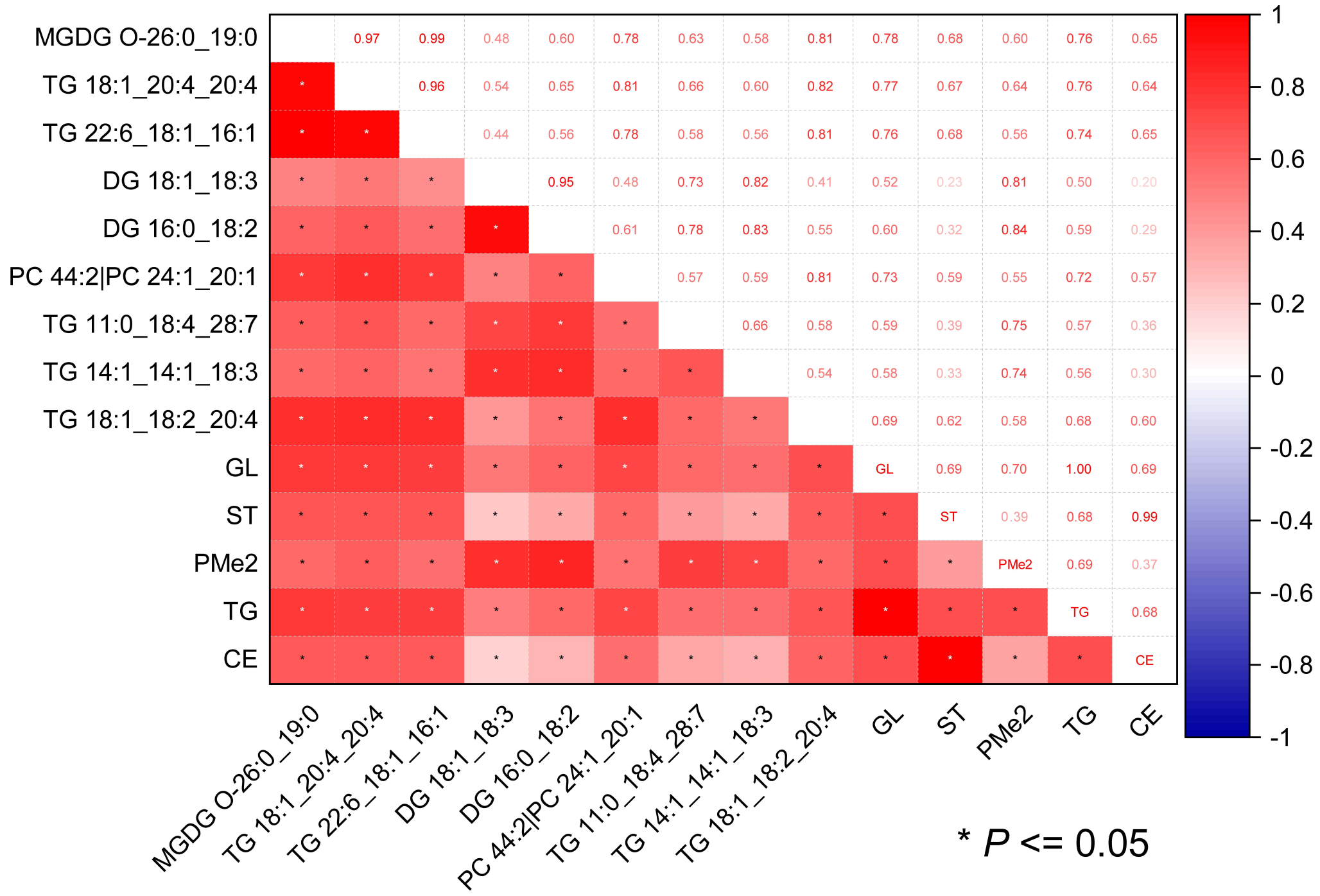

Figure S12. Correlation matrix between the nine prioritized lipids and lipid class/subclass traits associated with the chromosome 6 candidate locus. Asterisks indicate statistically significant correlations ($P < 0.05$).

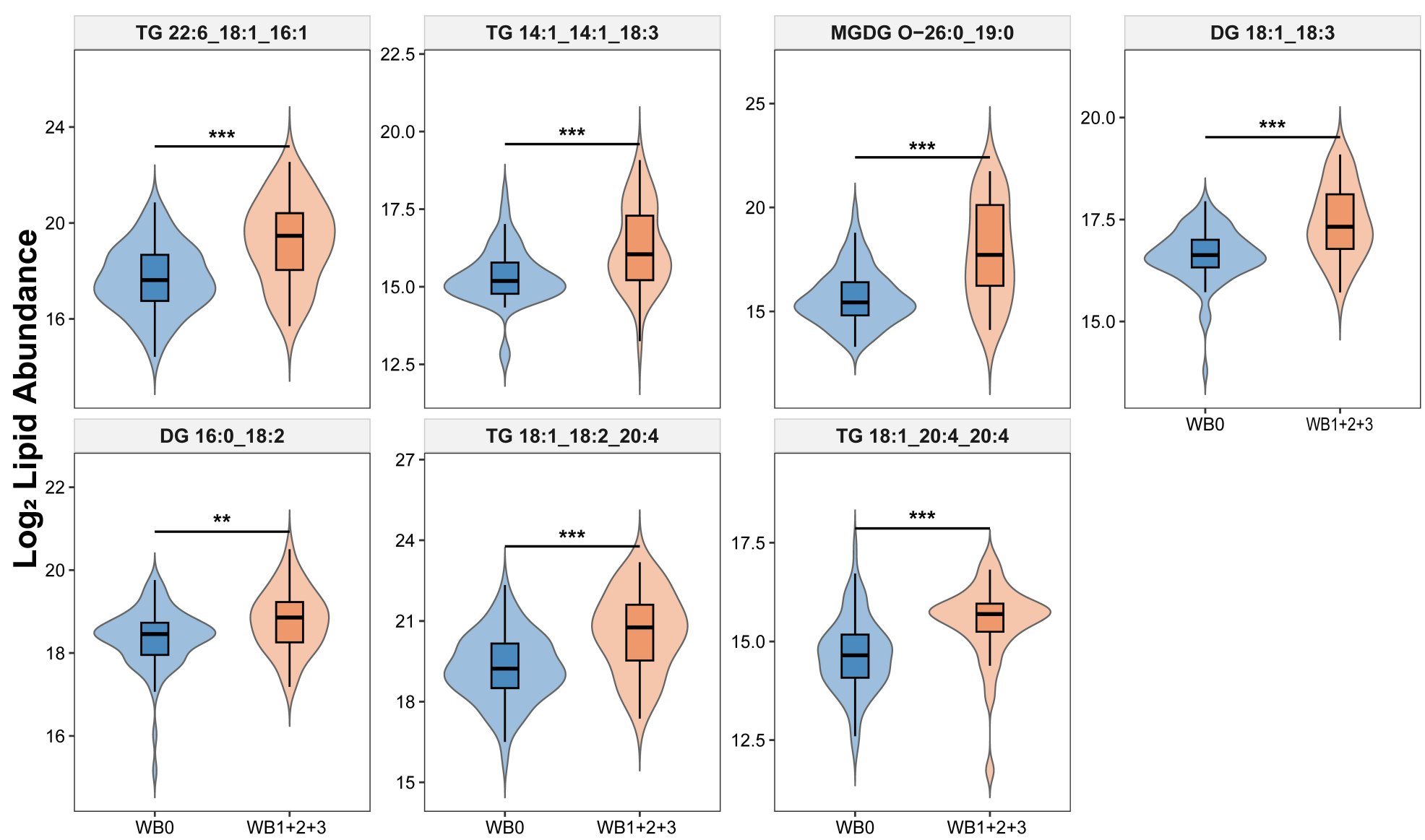

Figure S13. Validation of seven lipid biomarkers in the independent cohort. ** and *** indicate $P < 0.01$ and $P < 0.001$, respectively.

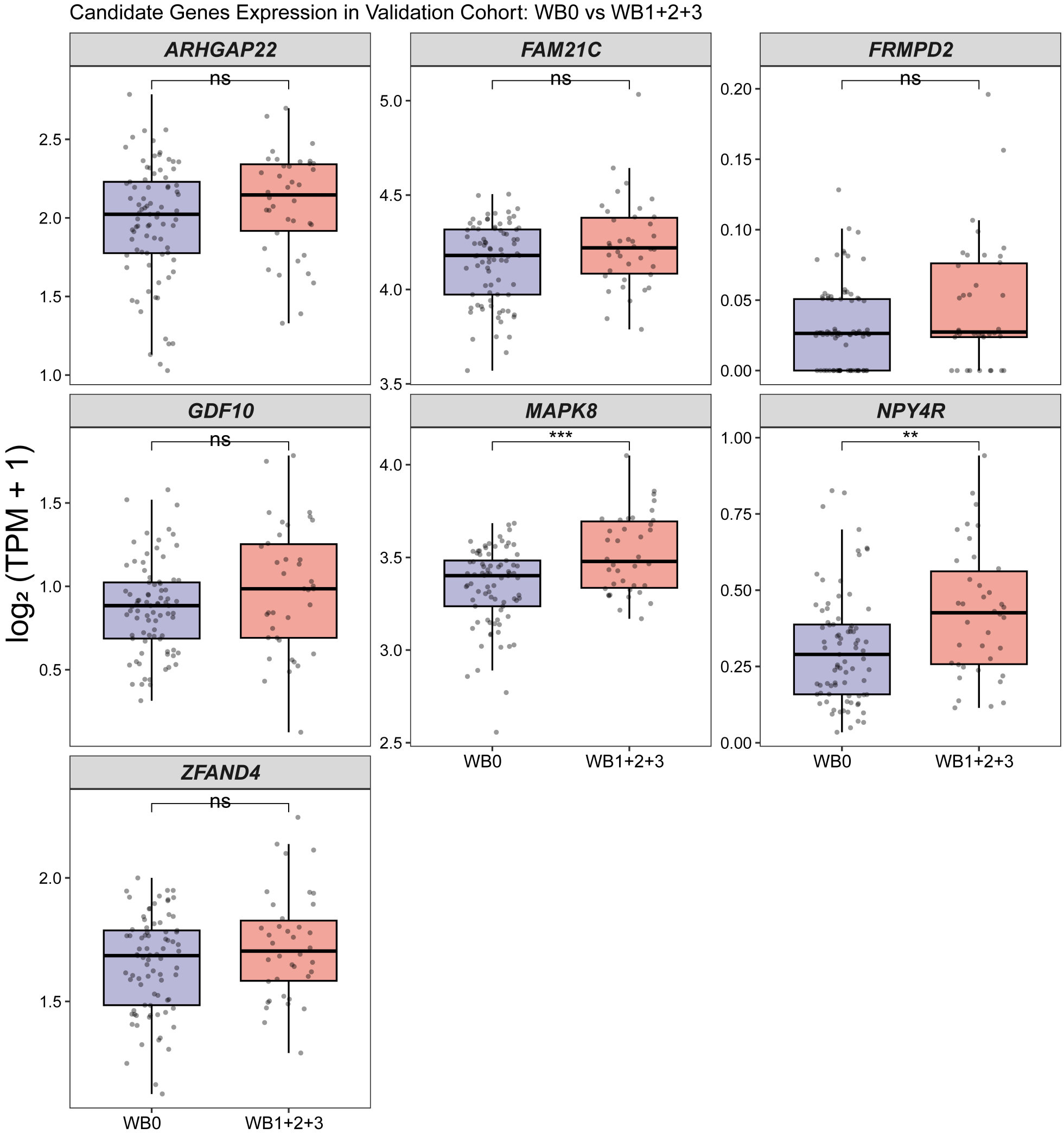

Figure S14. Expression validation of candidate genes in the independent cohort. ns indicates no significant difference, while ** and *** indicate $P < 0.01$ and $P < 0.001$, respectively.

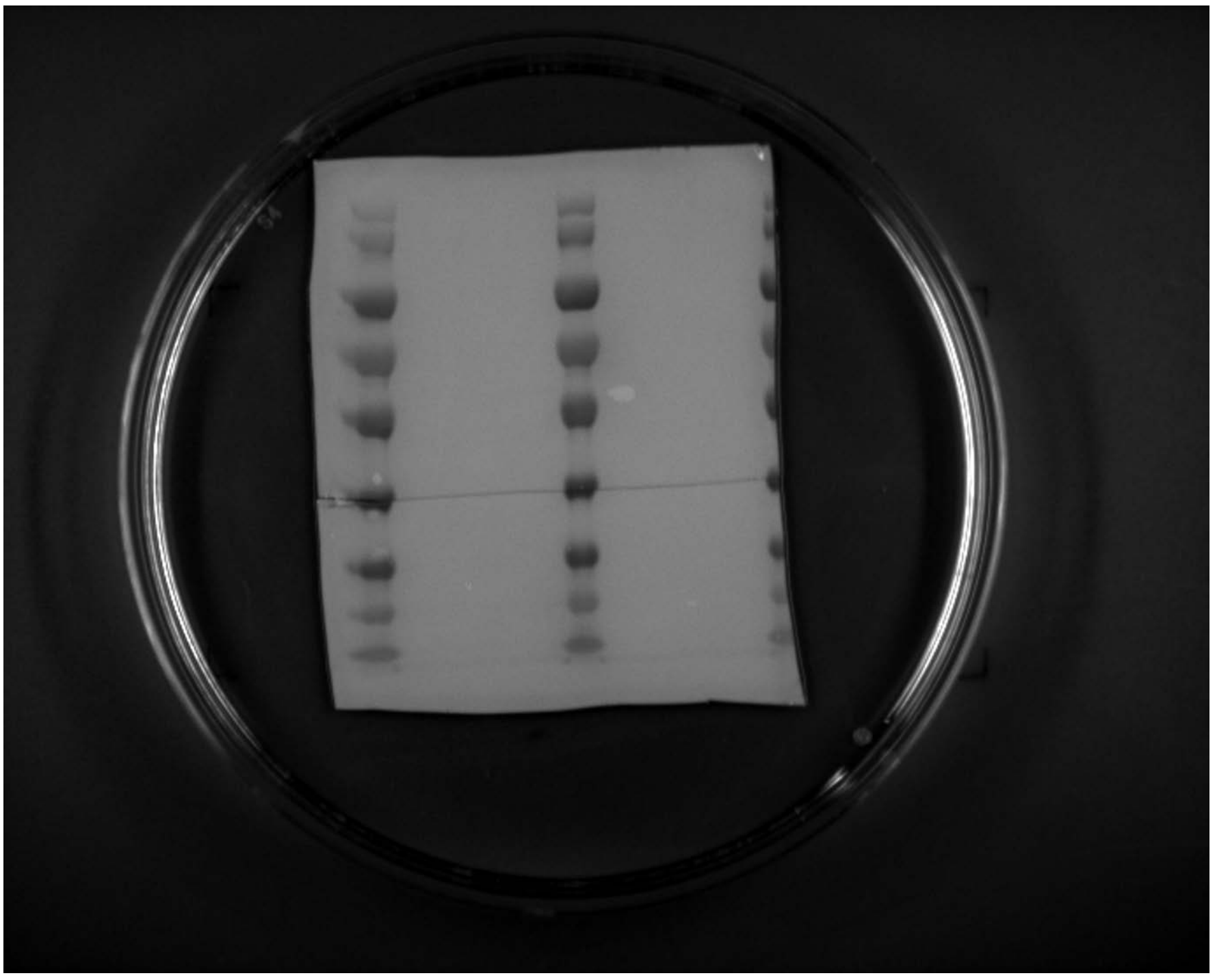

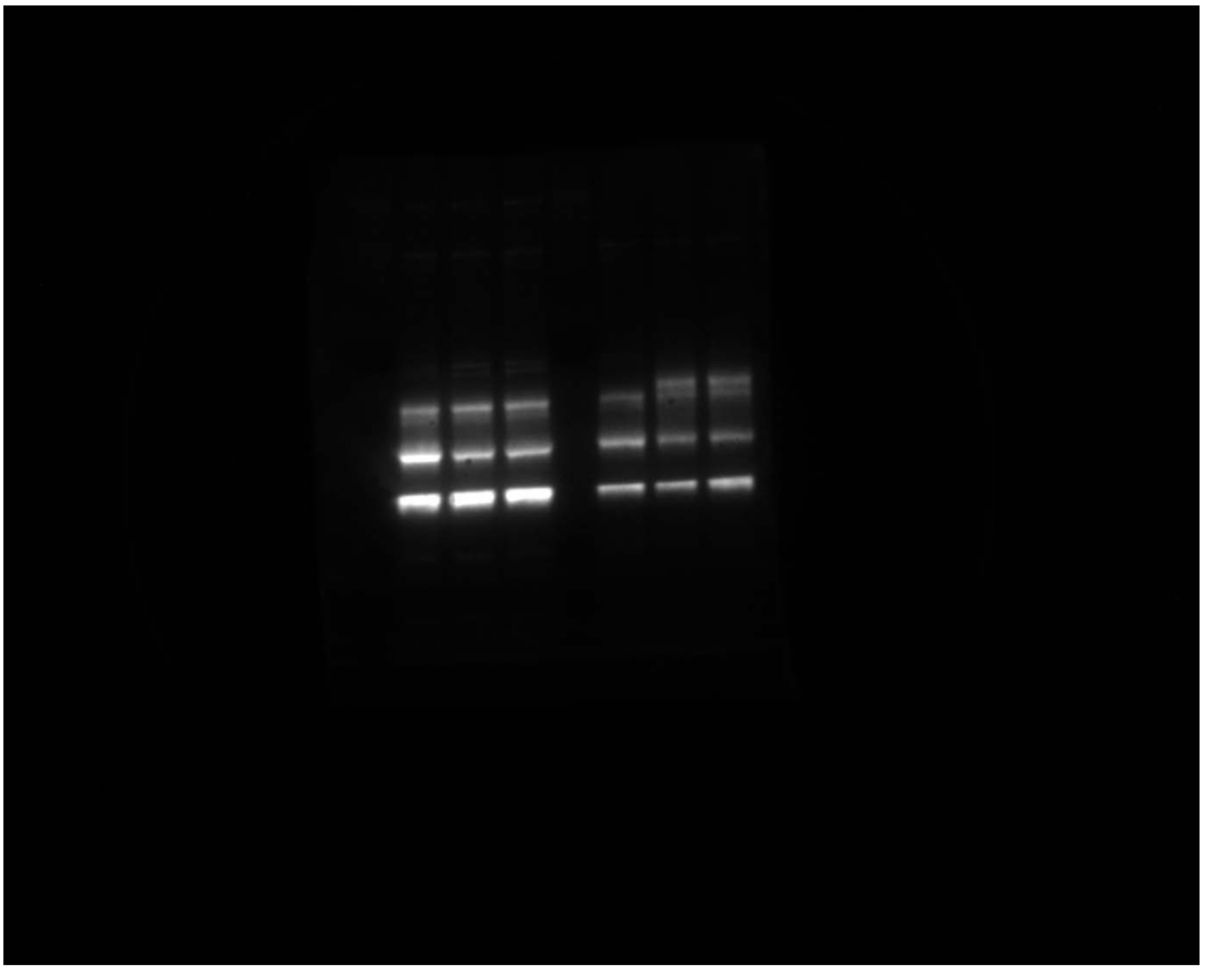

Figure S15. Original uncropped Western blot images for MAPK8 and GAPDH. The membrane was separated at the indicated position for antibody incubation and subsequently aligned for imaging. The original membrane orientation and relative positions of the bands were maintained.
